# The Anatomy to Genomics (ATG) Start Genetics medical school initiative: incorporating exome sequencing data from cadavers used for Anatomy instruction into the first year curriculum

**DOI:** 10.1186/s12920-016-0223-4

**Published:** 2016-10-06

**Authors:** Glenn S. Gerhard, Qunyan Jin, Barbara V. Paynton, Steven N. Popoff

**Affiliations:** 1 Lewis Katz School of Medicine at Temple University , Philadelphia, PA 19140 USA; 2 Department of Medical Genetics and Molecular Biochemistry, 960 Medical Education and Research Building (MERB), Lewis Katz School of Medicine at Temple University , 3500 N. Broad Street, Philadelphia, PA 19140 USA

**Keywords:** Exome sequencing, Medical education, Cadaver

## Abstract

**Background:**

The increasing use of next generation DNA sequencing in clinical medicine is exposing the need for more genetics education in physician training. We piloted an initiative to determine the feasibility of incorporating exome sequencing data generated from DNA obtained from cadavers used for teaching Anatomy into a first year medical student integrated block-style course.

**Methods:**

We optimized the procedure to obtain DNA for exome sequencing by comparing the quality and quantity of DNA isolated from several tissues by two different extraction methods. DNA was sequenced using exome capture and analyzed using standard methods. Single nucleotide variants (SNVs), as well as small insertions/deletions, with potential functional impact were selected by faculty for student teams to independently investigate and prepare presentations on their findings.

**Results:**

A total of seven cadaver DNAs were sequenced yielding high quality results. SNVs were identified that were associated, with known physical traits and disease susceptibility, as well as pharmacogenomic phenotypes. Students presented findings based on correlation with known clinical information about the cadavers’ diseases and traits.

**Conclusion:**

Exome sequencing of cadaver DNA is a useful tool to integrate Anatomy with Genetics and Biochemistry into a first year medical student core curriculum.

**Electronic supplementary material:**

The online version of this article (doi:10.1186/s12920-016-0223-4) contains supplementary material, which is available to authorized users.

## Introduction

A formal call for genomics education of physicians in anticipation of completion of the human genome project was made almost two decades ago [1]. This has been followed by more recent efforts to highlight this need [2], as well as analyses of why the overall state of knowledge of genomic medicine among physicians has been relatively limited outside of genetics specialists [3]. Despite the development of genetic and genomic educational resources for physicians [4], genomic medicine competes with other expanding areas of knowledge for the limited time available for continuing medical education. This has led to efforts to address education during residency training. For example, genomic medicine curricula have been developed for Pathology residency training by individual institutions [5] and the National Institutes of Health (NIH) has provided significant funding for the implementation of a genetics and genomics curriculum for residency programs in Pathology [6].

The need to enhance genomics education in medical school education has been recognized by professional organizations [7]. Several novel programs have been developed including analyzing single nucleotide polymorphism (SNP) genotyping of medical students’ own DNA [8, 9], and genomic medicine oriented tracks [9, 10]. Whole genome sequencing of medical student DNA has also been reported [11, 12]. Several years ago, Temple University School of Medicine also identified genetics as increasing in importance in the practice of medicine and began to implement changes to accommodate this need. This effort was formally inaugurated in late 2014 when the Department of Biochemistry was renamed the Department of Medical Genetics and Molecular Biochemistry. Concurrently, an opportunity to implement changes to the teaching of genetics arose as part of a major revision of the first year medical student curriculum in 2015. The Anatomy to Genomics ATG Start Genetics initiative was developed as an educational program whose primary objectives were to use genetics to better integrate and reinforce the teaching of Anatomy and Biochemistry, to expose the students to the potential medical application of next generation sequencing, and to provide a self-directed learning experience centered on genetics.

## Materials and methods

### Tissue selection and DNA isolation

Cadavers used for Anatomy instruction were embalmed using specialized procedures. Tissues were perfused with a 3:1 dilution of the Maryland State Anatomical Solution (Hydrol Chemical Company, Yeadon, PA) containing formaldehyde (2–3 %), methanol (24–28 %), phenol (22–26 %) and glycerine (38–42 %) pumped through the vascular system at high pressure via a femoral artery cannula. After allowing the fixative to fully permeate the tissues for a period of 24–48 h, the cadavers were stored at 4 °C for a period of time ranging from 6 to 18 months. Tissue samples for DNA analysis were obtained approximately 6 weeks after the onset of student dissection during which the cadavers are kept at room temperature. Sections of liver, skin, cardiac atrium and ventricle, and skeletal muscle were obtained for DNA analysis. Samples of tissue (1 cm^3^) were finely minced using a scalpel blade and then subjected to DNA isolation using either the QIAamp DNA FFPE Tissue Kit or the Qiagen DNeasy Blood & Tissue Kit (Qiagen, Inc.). For the FFPE procedure, extraction with xylene was omitted but incubation at 90 °C to reverse formalin crosslinking was performed. Both procedures included proteinase K digestion overnight at 56 °C. Samples treated with RNase (2 ul of 100 mg/ml of RNase) were incubated at 37 °C for two hours. For PCR, a 1069 bp fragment of the Carboxypeptidase 2 (CBP2) gene promoter was used with Accuprime Taq High Fidelity Taq Polymerase, and 35 cycles of 94 °C for 30 sec, 55 °C for 30 sec, and 68 °C for 1 min using forward primer sequence 5’- GTG CAA CCC TGT CTC TAC TAA A -3’ and reverse primer sequence 5’- TCT TGT TCC TGT GGG TCA ATC -3’.

### Exome sequencing

Exome library preparation was performed using the Human Exome Agilent v5 51Mbp (hEx-AV5) kit (Otogenetics, AL). DNA was sequenced on a HiSeq 2500 after exome capture using paired end reads of 100–125 bp with a designated average coverage of 30x or ~2.7-3 Gb. The data were then processed using the DNANexus platform consisting of BWA mapping + GATK SNP/Indel pipeline including mapping with BWA against human reference genome hg19/GRCh37, removing duplicates with Picard MarkDuplicates, realigning the mapped reads using GATK (2.3.9) with information of known indels from dbSNP and the 1000 Genomes project, recalibrating the base quality with GATK, and calling variants with GATK unifiedgenotyper followed by snpEff annotation. SNVs were computationally filtered for those classified as deleterious using the SIFT (Sorting Intolerant From Tolerant) tool [13] and/or those with a ClinVar clinical significance annotation [14] and included small insertions/deletions.

### SNV analysis

SNVs from each cadaver’s exome data classified as deleterious or with a ClinVar clinical significance annotation were selected by faculty (SNV lists for each cadaver contained in each clinical reasoning case in Additional file 1). Reference SNP ID numbers, or “rs” numbers, were provided for students to search in dbSNP [15] and OMIM (Online Mendelian Inheritance in Man; An Online Catalog of Human Genes and Genetic Disorders) [16]. Step by step search instructions were provided, including screen shots of expected results for an example SNV (Additional file 1). The students were to determine the amino acid substitution for each SNV along with biochemical classification of amino acid substitutions. They were also instructed to compose a PowerPoint presentation with the primary goal of explaining how the SNVs found through exome sequencing may have been related to the traits and diseases the cadaver may have experienced. For most cadavers, cause of death and limited aspects of the past medical history such as major diagnoses or surgeries were provided to the students by faculty as a standard part of the Anatomy course. In addition, observations made during dissection were also discussed as a standard part of the Anatomy course.

## Results

### DNA isolation

**Fig. 1:**
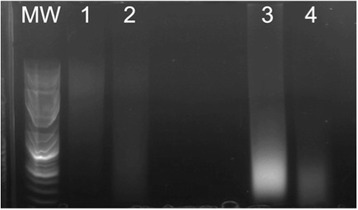
Agarose gel electrophoresis of DNA isolated from cadaver tissues. MW = GeneRuler 1 kb Plus DNA Ladder (Thermo Fisher Scientific). Lane 1: Heart DNA extracted with DNeasy Blood & Tissue Kit. Lane 2: Liver DNA extracted with DNeasy Blood & Tissue Kit. Lane 3: Heart DNA extracted with FFPE kit. Lane 4: Liver DNA extracted with FFPE kit

DNA was first isolated from liver and cardiac tissue as two relatively high DNA content tissues. The initial protocol according to the manufacturer yielded qualitatively low DNA concentrations using the DNeasy Blood & Tissue Kit (Fig. 1 lanes 1 and 2) that was quantitatively verified (Table 1). We then tried the QIAamp DNA FFPE Tissue Kit (Fig. 1 lanes 3 and 4), which, despite higher concentrations (Table 2), resulted in lower molecular weight nucleic acid consisting possibly of RNA and/or degraded DNA.

**Table 1:** DNA concentration and quality corresponding to lanes described in Fig. 1

Tissue	DNA (ng/ul)	260/280 nm
1. Heart	21.0	1.69
2. Liver	39.0	1.84
3. Heart	114.6	1.86
4. Liver	61.9	1.87

**Table 2:** DNA concentration and quality corresponding to lanes described in Fig. 2

Tissue	DNA (ng/ul)	260/280 nm
1. Liver	448.0	2.04
2. Heart	246.9	1.90
3. Muscle	88.0	1.90
4. Skin	28.7	1.86

**Fig. 2:**
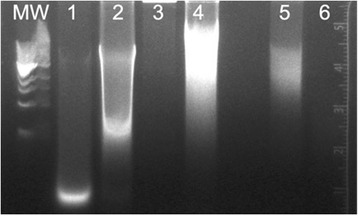
Agarose gel electrophoresis of DNA isolated from cadaver tissues using the DNeasy Blood & Tissue Kit. MW = Quick-load 1 kb DNA Ladder (New England BioLabs). Lane 1: Liver without RNAse treatment. Lane 2: Heart without RNAse treatment. Lane 3: Liver with RNAse treatment. Lane 4: Heart with RNAse treatment. Lane 5: Skeletal Muscle with RNAse treatment. Lane 6: Skin with RNAse treatment

Based on these initial results, we then increased the amount of tissue lysate applied to the DNA isolation column to the maximum recommended, omitted the 90 °C FFPE step, obtained cadaver skeletal muscle and skin samples as other tissue sources of DNA, and, in order to rule out the presence of RNA, treated with RNase. We found that both liver and heart had substantial amounts of RNA that could be removed following treatment with RNase (Fig. 2). Following RNA removal, heart provided a robust amount of high molecular weight DNA, much greater than skeletal muscle, skin, or liver. We then determined whether the DNA could be used for a PCR reaction (Additional file 1: Figure S1). A correctly sized PCR product (~1 kb) was obtained with heart DNA treated with RNase. After these initial experiments to optimize recovery of high quality/quantity DNA, we isolated DNA from seven cadaver heart samples obtained by the medical student dissection teams to analyze via exome sequencing.

### Cadaver selection

The first year medical student class at the Temple University Lewis Katz School of Medicine is composed of 210 students organized into seven groups or colleges of 30 students each, with each college composed of cadaver dissection teams of six students. Each of the 35 dissection teams is assigned a cadaver in the Human Anatomy and Development course (Block 1) of the main Fundamentals of Medicine course. A one hour introductory didactic session (outline in Additional file 1) was held with the entire first year medical school class to describe the ATG initiative, timeline (Additional file 1) and expectations, and to answer any questions. The cadaver dissection teams were assigned the task of obtaining heart muscle samples from each cadaver near the end of the dissection section of the course after having completed dissection of the back, limbs, thorax and abdominopelvic cavity. Students from each of the seven colleges also met as a group to compose a prioritized list for sequencing of the five cadavers within each college (Additional file 1) based on information available regarding the cause of death and their anatomic/pathological findings that were made during the dissection.

The students’ top priority was selected for exome sequencing in four of the colleges (Table 3). The second priority was used in one college because the cause of death was gastric cancer, which was the top selection by another college. Breast cancer was selected over multiple myeloma, and lung cancer over liver cancer, which was considered metastatic with an unknown primary. DNA samples obtained from heart ventricle from each of the seven cadavers were analyzed by exome sequencing.

**Table 3:** Student priority and cause of death of cadavers sequenced

Student priority	Cause of Death/Clinical Information
2	Breast Cancer and Pacemaker
2	Dementia
1	ALS
1	Gastric Cancer, Metastases
1	Prostate Cancer/Radical Prostatectomy/Metastases
2	Lung cancer
1	Myelodysplastic Syndrome

**Table 4:** Example set of SNVs selected from cadaver with diagnosis of Myelodysplastic syndrome

Chr	dbSNP	Gene	1000G MAF	ClinVar Significance	ClinVar Annotation
5	rs61748181	*TERT*	0.014	Pathogenic	Aplastic_anemia
17	rs1042522	*TP53*	0.602	Benign	Codon 72 polymorphism/neoplastic syndromes/hereditary
22	rs4680	*COMT*	0.389	Benign	Catechol-o-methyltransferase polymorphism
22	rs1065852	*CYP2D6*	0.255	Pathogenic	Poor metabolism of Debrisoquine
7	rs10246939	*TAS2R38*	0.549	Pathogenic	Phenylthiocarbamide tasting
5	rs16891982	*SLC45A2*	0.441	-	Skin/hair/eye pigmentation variation
11	rs1126809	*TYR*	0.112	Pathogenic	Waardenburg syndrome and ocular albinism
16	rs1805007	*MC1R*	0.030	Pathogenic	Red hair/fair skin/Increased analgesia from kappa-opioid receptor agonist
12	rs41276738	*VWF*	0.001	Pathogenic	Von Willebrand disease
1	rs6025	*F5*	0.994	-	Thrombophilia due to factor V Leiden

Please see Additional file 1 for chromosomal position, reference and alternative alleles, and depth of coverage

### Exome sequencing

Due to the short period of time from the harvesting of tissue at the end of Block 1 to the end of Block 2 when workshop presentations were scheduled, we partnered with a commercial service who could accomodate the compressed schedule for data generation for exome sequencing (Otogenetics, AL). DNA quality control was conducted and all samples passed acceptable standards. HiSeq sequencing was performed using the Agilent v5 51Mbp exon capture kit with a target mean coverage of 30X (3 GB of data). The number of base pairs and reads generated was consistent with this target (Additional file 1: Table S1). All samples passed quality control basic statistics per FastQC report, and per sequence quality scores, per base sequence content, per base GC content, sequence length distribution, and overrepresented sequence were above QC thresholds (representative FastQC report shown in Additional file 1).

### Workshop presentations

The first year Block 2 medical curriculum is organized into didactic lectures and “Clinical Reasoning Cases” or workshops. Two different workshop formats were used. In the informal format, the class was divided into eight sections composed of the six student teams. Each team was allotted 20 min to complete researching a structured clinical case illustrating specific medical genetic and/or biochemical topics provided to them a week or more prior, followed by selection of one team to discuss the case in a 10 min “chalk talk”, or more accurately inkboard, presentation. In the formal workshop format, all teams prepared oral Power Point presentations during the first 20 min allotted, with one team selected to give a presentation in the last 10 min. We opted for the formal workshop format for which the students had to present oral PowerPoint presentations, except that all presentations had to be completed before the workshop session and the presentations were 15 min in length. Teams that were selected to present were not announced until the time of presentation so that all groups were prepared in case they were selected. The presentation time included several minutes for questions and discussion.

### Didactic presentations

Two formal didactic presentations were held with attendance on a voluntary basis. The material presented included preparation and analysis of DNA, a primer on DNA sequencing, and results of DNA analysis from the cadaver samples (presentation outline in Additional file 1). The second presentation discussed bioinformatics analysis and exome sequencing (presentation outline in Additional file 1). Both presentation files were uploaded to the Temple University LCMS+ curriculum portal for use in composing workshop presentations.

### Exome sequencing results and selection of SNVs

The total number of single nucleotide variants with a deleterious SIFT score and/or a ClinVar clinical significance annotation found per cadaver DNA ranged from 1977 to 2752. SNVs predicted to cause essentially all major types of effects on gene expression and function were found including missense, stop loss, stop gain, splice acceptor, splice donor, frameshift, premature start codon gain, five prime UTR, three prime UTR. SNVs from each cadaver’s exome results that. were potentially related to the cadaver’s cause of death or related findings from dissection and traits such as skin or hair color as well as pharmacogenomics variants, variants with very low allele frequency, or common variants in genes associated with clinical conditions relevant to the cadaver were selected by faculty and provided to the students for the workshop presentations.

### Selected SNVs

Approximately ten SNVs selected for each of the seven cadavers sequenced (contained in each Clinical Reasoning Case in Additional file 1; example shown in Table 4) were provided to the five teams from each College that prioritized the cadavers selected for sequencing, thus all five teams from each of the seven Colleges had the same exome results from which to prepare PowerPoint presentations. Exome sequencing identified several variants (genomic coordinates contained in each Clinical Reasoning Case in Additional file 1) in genes that were previously reported to be associated with several cadaver phenotypes, including a variant in the *TARDBP* gene in a cadaver who died of amyotrophic lateral sclerosis (ALS) [17], a common missense SNV in the *MSR1* gene associated with prostate cancer through GWAS [18] in a cadaver who had undergone a radical prostatectomy, a common variant in the *PRNP* gene previously associated through a GWAS meta-analysis with Alzheimer’s disease [19] in a cadaver with a history of dementia, and SNVs in *F5* (Leiden) [20], *HFE* (C282Y) [21], and von Willebrand factor (*vWF*) [22].

### Workshop presentations

PowerPoint presentations were prepared by all 35 college teams. Over a period of two hours, a total of eight teams presented their cadaver’s findings with 15 min allocated for each presentation. Due to the requirement that all six members of a team participate in the presentation, and concern over the inexperience of the students at developing presentations within the 15 min allotted, the students were advised to focus the efforts on the associations of the SNVs and genes with traits and diseases. Some teams did incorporate information on DNA preparation, quality and background information on exome sequencing, and expected or known biochemical effects of the SNVs on protein function, while others did not. All teams did provide information on the genes associated with the SNVs, the global minor allele frequencies of SNVs, associations of the SNVs and genes with traits and diseases, how the SNVs may have impacted the traits and diseases the cadaver may have experienced, and how the SNVs may have impacted subsequent health care, in addition to references and resources. Examples of two of the teams’ presentations are included in the Additional file 1.

## Discussion

A survey conducted in 2013–2014 examined genetics curricula in US and Canadian medical schools to determine how advances in genomic technologies were affecting educational trends [23]. Similar to Temple, the majority of genetics was formally taught during the first year. A majority of respondents had increased the degree of integration of genetics with the remainder of the medical curricula, most transitioning to a block or integrated type of structure. However, the majority of survey participants felt that the amount of time devoted to genetics instruction was insufficient. Temple had already undergone the transition to a block structure, but was in the process of further revising the first two blocks of the year 1 major Fundamentals of Medicine course to increase the amount of active learning methods and to better integrate the blocks. The disciplinary blocks of the Fundamentals of Medicine course began with Anatomy in Block 1 followed by Block 2, which was further organized into a Biochemistry, Molecular Biology and Genetics section and a Cell Biology, Cell Physiology, Microstructure, Microbiota and Biostatistics section. Block 2 was the focus of curricular revision, providing an institutionally supported opportunity to revise the genetics curriculum.

The major revision to the Block 2 curriculum was to decrease the number of didactic hours, increase the clinical relevance of workshops, and increase self-directed learning experiences. To achieve these goals, we first developed a formal curriculum using the Association of Professors in Human and Medical Genetics (APHMG) recently updated medical school core curriculum in genetics [23] as a guide with modifications that reflected the need to integrate with other course content and that included clinical and translational topics. This core genetics curriculum provided the framework for the revised content of didactic presentations and the re-design of workshops. The Anatomy to Genomics ATG Start Genetics initiative was also implemented as a self-directed learning experience in a workshop format.

The first major issue we had to address was whether DNA that was of sufficient quality to undergo exome sequencing could be obtained from cadaver tissue. The process by which individuals donate their bodies through anatomical gift programs allows for variable post-mortem intervals and storage temperatures. In addition, there are no recognized standards for embalming techniques [24]. Formaldehyde-based methods predominate, despite the existence of alternative methods [25]. Periods of formalin fixation longer than 4–8 weeks have been reported to result in substantial loss of integrity of DNA even for conducting simple polymerase chain reaction (PCR)-based single nucleotide variant assays [26, 27]. However, the anatomical solution that was used for preservation of cadavers consisted of a formula that minimized the requirement for higher concentrations of formaldehyde used in traditional embalming solutions and likely helped to maintain DNA integrity. Based on the discouraging results of initial experiments, we recognized that the amount of starting tissue had to be greatly increased. The ability to consistently obtain sufficient amounts of relatively high quality genomic DNA for exome sequencing enabled us to proceed with the rest of the initiative.

We considered whether to conduct laboratory-based sessions on DNA isolation, library preparation and sequencing, and bioinformatics analysis. However, with 210 first year medical students, the logistics necessary to accommodate such a large number were not trivial. In addition, we did not want to further add complexity and burden to the ongoing efforts to revise the curriculum. We thus opted to cover these topics using didactic lectures. The lectures were offered as an optional activity due to the compression of the didactic portions of the curriculum and the competition for the available time slots. The trend in medical education away from lectures to case-based formats has been driven in part by the ready availability of alternative learning modalities, including video capture of lectures [28, 29]. We thus made the video capture of the lectures and the PowerPoint presentation files available to all students through the on-line curriculum portal.

Although we did not conduct a hands-on session on the bioinformatics pipeline used to identify SNVs from the raw sequence data, we did have as a goal that every first year medical student would gain experience in searching dbSNP and OMIM as a form of clinically relevant bioinformatics instruction [30]. A step-by-step guide was provided, similar to that described for a medical student curriculum into which OMIM had been integrated [31], such that the two databases were used as starting points to provide information on the SNVs found by exome sequencing. Feedback from students indicated that they were interested in expanding this training and including more hands-on instruction.

Several previous efforts to increase genetics and genomics education in the undergraduate and graduate medical curricula have been reported [5, 10, 32]. Genetics and genomics “tracks” have also been developed for medical students [33, 34]. Several schools have used medical students’ own genotyping or sequencing data for teaching. Genotyping medical students using commercial panels of 300–600 K SNPs [35, 36] appears to increase knowledge of genetics with little regret or anxiety [35, 36], although does carry a number of ethical, legal, and social issues [37]. Personal results of whole genome sequencing [11], while increasing motivation to learn, was found to cause distress and regret in some students [12], although only five of the students who received their own whole genome sequencing results were MD or MD/PhD students who had self-selected into sequencing. It may be expected that an even wider range of both positive and negative effects from personal sequencing may be expected given the diverse backgrounds and knowledge in genetics and genomics that is present in a typical medical school class. An informal in-lecture survey of the first year class during the ATG Start Genetics initiative indicated that most, but not all, would be willing to have their DNA sequenced if it were provided free of charge, but many expressed concerns over privacy.

We thus chose to use cadaver DNA for exome sequencing, rather than the students’ own DNA, in part to expose every member of a first year class to next generation sequencing and to avoid any of the negative consequences of personal genotyping or sequencing. Other institutions have used whole genome sequencing of Anatomy cadaver DNA [38], but restricted participation to primarily combined M.D./Ph.D. students from a Medical Scientist Training Program in combination with histopathological examination of the cadaver’s tissues by a pathologist. We sought to engage the entire medical school class, thus we chose to use exome sequencing, which is more economical and medically relevant at this point. We also wanted to develop a process that would minimize additional manpower needed to teach the first year curriculum. Due to the wide variation in educational and training backgrounds across a class of 210 medical students, in contrast to a small number of Medical Scientist Training Program trainees, we also chose to pre-select variants for the students to interpret and to present the exome analysis process didactically, rather than try to engage the students in the analysis. This was also more feasible to implement in the first part of the first year curriculum, in which there are many competing demands. However, we foresee transitioning from exome to whole genome sequencing as costs come down and clinical utility increase, as well as offering more in-depth experiences in laboratory and bioinformatics aspects of exome sequencing.

During the initiative, several students inquired about ethical issues surrounding the use of cadaver DNA, which is not considered human subjects research, and is an under-addressed topic that has only recently been discussed in any detail [39, 40] and which we have also recently addressed [41]. Another ethical issue that arose was illuminated by the finding of the *PRNP* variant in a cadaver with dementia. Although the SNV found was a common variant, it raised the possibility of finding a rare pathogenic *PRNP* SNV with the potential for disease transmission. Despite the use of fixation during embalming, microbial agents, including viruses, bacteria, and prions can remain infective in cadavers [42]. Next generation sequencing has the potential for identifying germline prion mutations as well as passenger DNA from microbes. We plan to further develop the ethical, legal, and social issues pertaining to cadaver sequencing raised during the pilot experience as part of the curriculum going forward.

Another disadvantage of genotyping students, whose average age is likely less than 25, is that the number of disease related findings will be be minimal yet incidental findings may be found. Using cadaver DNA allowed for both the association of variants with an underlying disease process as well as the potential to identify incidental findings, such as pharmacogenomics variants, in addition to variants for Mendelian disease, e.g., *HFE* C282Y. SNVs related to skin and hair color could also be correlated with both the physical observations of the cadaver and related to biochemical pathways, particularly tyrosine metabolism, a difficult area of biochemistry education.

As we have recently advocated [43] we believe that genomic medicine should be regarded fundamental a discipline as Anatomy for beginning medical students. We hope to extend genomic medicine across the entire 4-year medical school curriculum, as well as into graduate and continuing medical education. With these efforts we hope to contribute to helping medical education keep pace with genomic and precision medicine [44].

## Additional file

Additional file 1: Supplmentary Information. (PDF 4086 kb)

## Abbreviations

ALS: Amyotrophic lateral sclerosis
CBP2: Carboxypeptidase 2
OMIM: Online Mendelian Inheritance in Man; An Online Catalog of Human Genes and Genetic Disorders
SNP: Single nucleotide polymorphism
SNVs: Single nucleotide variants
SNVs: Single nucleotide variants
*vWF*: Willebrand factor
