# Supplementary material for: The Anatomy to Genomics (ATG) Start Genetics medical school initiative: incorporating exome sequencing data from cadavers used for Anatomy instruction into the first year curriculum
Source: BMC Med Genomics. 2016 Oct 6;9:62. doi: 10.1186/s12920-016-0223-4 (PMC5053090; doi:10.1186/s12920-016-0223-4)
Supplement: Additional file 1: — Supplmentary Information. (PDF 4086 kb) [file 12920_2016_223_MOESM1_ESM.pdf]

Additional file 1

The Anatomy to Genomics (ATG) Start Genetics medical school initiative: incorporating exome sequencing data from cadavers used for Anatomy instruction into the first year curriculum.

Gerhard, et al.

1. Cadaver exome sequencing workshop description and timeline
2. Cadaver prioritization
3. Outline of DNA isolation/Exome sequencing process didactic
4. Outline of Exome data analysis didactic
5. Clinical Reasoning Case Instructions
6. Clinical Reasoning Cases 1-7
7. Table of amount of basepairs and reads generated by exome sequencing of cadaver DNA
8. Representative FastQC report for exome sequencing
9. PCR of cadaver heart DNA
10. Example Medical Student Exome Sequencing PowerPoint Presentation 1
11. Example Medical Student Exome Sequencing PowerPoint Presentation 2

1. Cadaver exome sequencing workshop description and timeline

Description: The Departments of Medical Genetics and Molecular Biochemistry (MG & MB) and Anatomy and Cell Biology have partnered to incorporate modern Medical Genetics into the Temple School of Medicine curriculum through next-generation sequencing of DNA obtained from cadavers used for teaching Anatomy. This learning activity is designed to promote student participation in group learning and is a "Self-Directed Learning" experience. It involves *self-assessment of learning needs; independent identification, analysis, and synthesis of relevant information; and appraisal of the credibility of information sources".* Self-directed learning experiences and time for independent study allow students to develop skills of lifelong learning.

Objectives: By the end of the course, students should be able to:

1. Describe the process of DNA isolation and next-generation sequencing (NGS) data generation.
2. Describe the process of NGS data analysis.
3. Describe the benefits and limitations of integrative genomic analyses for patients with inherited and common diseases.
4. Use online tools to interpret the clinical significance of genomic data.

Schedule:

1. **Sample collection and cadaver selection (Block 1 Activity)**
2. **DNA isolation/Exome sequencing presentation (Voluntary Learning Activity)**
3. **Exome data analysis presentation (Voluntary Learning Activity)**
4. **Presentations of cadaver sequencing results (Block 2 Workshop)**

Timeline:

Week	Activity
0	Start Anatomy
6	Prioritize Cadavers/Harvest Tissue
8	DNA isolated for sequencing
9	DNA Didactic
10	Exome Sequencing Data Generated
12	Exome Didactic
13	Selected SNVs Provided to Students
16	Oral Presentations

2. Cadaver prioritization

Each cadaver dissection group selected a cardiac muscle sample from their cadaver for DNA isolation. Each doctoring group met to compare their findings and prioritized their cadavers for exome sequencing (below). The cadavers sent for exome sequencing are in **bold**.

Nelson College:

1. Table 8: Multiple Myeloma
2. **Table 16: Breast Cancer and Pacemaker**
3. Table 14: Pulm. and Cardiac Issues, obese
4. Table 6: Pulmonary Hypoxia (different aortic branching)
5. Table 22: No obvious pathologies

Saunders College

1. Table 32 - Metastatic Gastric Cancer
2. **Table 38 - Dementia**
3. Table 40 - Metastatic Lung Cancer
4. Table 24 - Respiratory Arrest
5. Table 30 - Natural Causes

Sherry College

1. **Table 35 - ALS**
2. Table 34 - Leukemia
3. Table 36 - Hip complications and old age 101
4. Table 33 - Respiratory arrest
5. Table 37 – COPD

Babcock College

1. **Table 3; Gastric Cancer, Metastses**
2. Table 1; Stroke, Enlarged Heart, 3 way CABG
3. Table 4; Prostrate cancer but little findings in body
4. Table 2; Natural Causes, No Pathology
5. Table 5; No Pathology

Marks College

1. **Table 26; Prostate Cancer/Radical Prostatectomy/Metastases**
2. Table 28; Not provided
3. Table 29; Not provided
4. Table 27; Not provided
5. Table 25; Not provided

Durant College

1. Table 19: Liver Cancer and Esophageal and Lung Tumors

2. **Table 20: Lung cancer**
3. Table 18: Hiatal Hernia
4. Table 21: Aortic Aneurysm possibly
5. Table 17: Nothing of Interest

<u>Parkinson College</u>

1. **Table 12 - Myelodysplastic syndrome**
2. Table 09 - Pancreatic Cancer
3. Table 11 - Vascular Dementia
4. Table13 - Found a device (Greenfield filter) in IVC
5. Table 10 - Natural Causes

3. Outline of DNA isolation/Exome sequencing process didactic presentation

The outline of a 1 hour presentation on the isolation of DNA from the cadaver samples and the subsequent exome sequencing process is shown below.

Cadaver DNA and Sequencing Process
- Nucleic Acid Preparation- Sample Source
- Basic Steps in Isolating DNA from Blood
- DNA Isolation Methods Solid Phase Procedures
- DNA Isolation Method Comparison
- Assess DNA Quantity by UV Spectrophotometry or Gel Electrophoresis
- Cadaver DNA Isolation Results
- DiDeoxy (Sanger) Sequencing
- Illumina Next Generation Sequencing
- Conversion of images to bases
- Next Gen Sequence Data Amount and Transport
- What’s next for the cadaver heart DNA?

4. Outline of exome data analysis didactic presentation

An outline of the didactic presentation for analyzing next generation sequencing data offered prior to the workshop session is shown below.

Cadaver Exome Analysis

- Exome variants
- Disadvantages of exome sequencing
- Broad Next Generation Sequencing Data “Pipeline”
- “Raw data”- FastQ files
- SAM (Sequence Alignment/Map) and BAM (binary version of a SAM) format
- Bam files
- “Pipeline”
    - Mapping and alignment (BAM file)
    - QC
    - Coverage
    - sequence variation (SNPs/Indel report (.VCF file)
    - vcf annotations
    - functional prediction
    - population frequency
- Bioinformatics Analysis Output
- Types of variants found
- PolyPhen/SIFT pathogenicity prediction
- Examples of Exome sequencing
    - SOD1 D90A Gene Mutation
    - SOD1 D90A Sanger Sequence Confirmation
    - NEJM HABP2
- Ethical Dilemmas of Whole Genome Sequencing

5. Clinical Reasoning Case Instructions

EACH GROUP WILL PREPARE A 15 MINUTE POWER POINT PRESENTATION TO BE PRESENTED AT THE SCHEDULED WORKSHOP (PRESENTATIONS MUST BE FINISHED PRIOR TO THE WORKSHOP!) FOCUSING ON THE RESULTS AND CONCLUSIONS DERIVED FROM EXOME SEQUENCING OF THE CADAVER DNA. In the presentation, you should discuss/review background information concerning the purpose/clinical utility of sequencing, the methodology used, the results from your analysis of the sequencing data, and any conclusions regarding the anomalies/pathologies observed in the cadaver that was selected for sequencing. Specific points that should be addressed in your presentation include DNA preparation and quality, genes associated with the variants, significance of mean allele frequency, advantages and disadvantages of exome sequencing, expected or known biochemical effects of the variants on protein function, associations of the variants and genes with traits and diseases, background information on the associated diseases and traits, and how the variants may have impacted the traits and diseases the cadaver may have experienced and how that may have impacted subsequent health care. Don't forget to list the references and resources used to guide you through this exercise and to prepare your presentation.

The primary results from the exome sequencing for each of the selected cadavers have been incorporated into the workshop cases below.

Clinical Reasoning Case Instructions

Approximately 10 variants found by exome sequencing were selected for each cadaver and listed in a table for each doctoring college. The results are different for each cadaver! Each of the variants should be classified by type and how they would impact the protein or RNA. An example for the Sickle Cell Disease hemoglobin beta (HBB) gene variant is shown below. For each variant, this information should be listed in your presentation. Following this information, genes that could be hypothesized to be associated with the phenotype based on current knowledge of gene function, pathway, expression pattern and support for why and how the genes and variants may have played a role in the cadaver's health and health care. If the cadaver were alive, this could represent a diagnostic test.

SNV rs334 has a global Mean Allele Frequency of 0.0274 (2.74%) and causes a missense Glu7Val substitution in the hemoglobin beta (HBB) gene resulting in an acidic to non-polar amino acid substitution.

First, take the "rs" number that is found in the variant table dbSNP column and enter into the Search Entrez dbSNP database search field:

(http://www.ncbi.nlm.nih.gov/projects/SNP/

For the Sickle cell variant, the "rs" number is rs334.

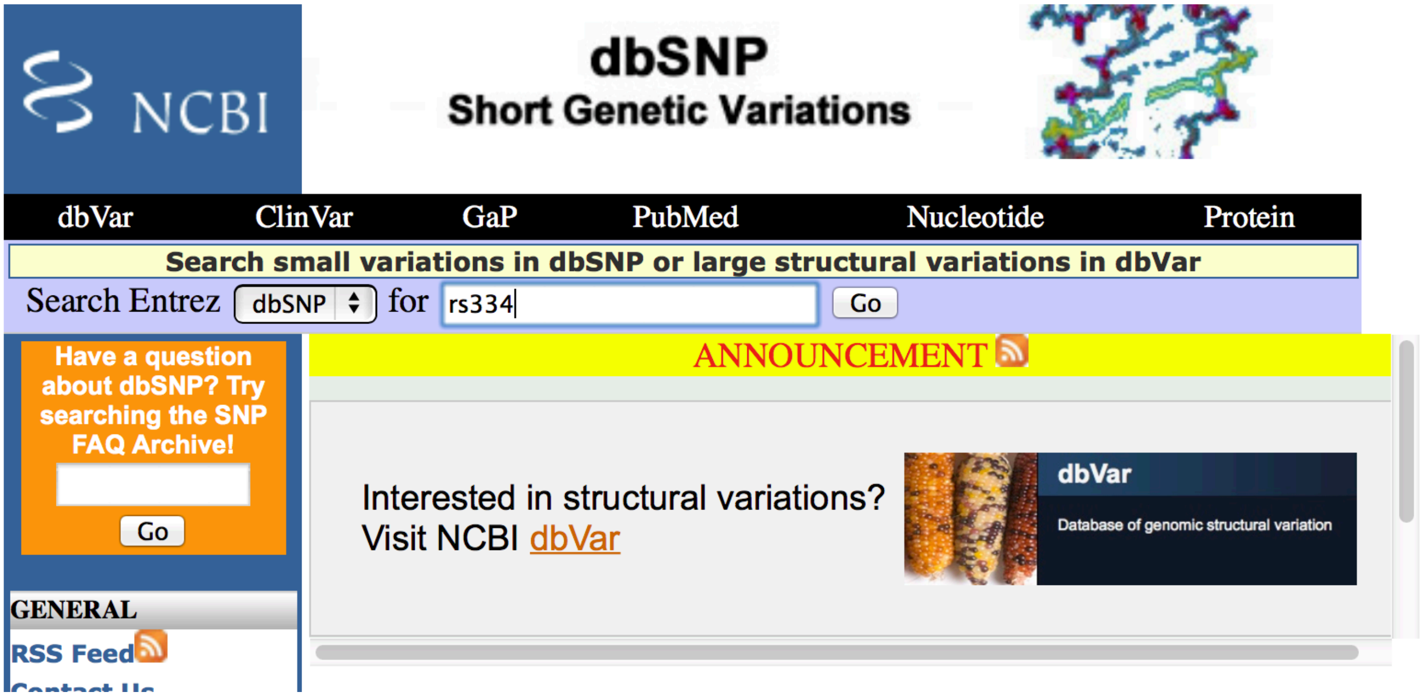

The results for rs334 should appear as:

Results: 4

1. rs334 *[Homo sapiens]*

GACACCATGGTGCATCTGACTCCTG[A/C/G/T]GGAGAAGTCTGCCGTTACTGCCCTG

Chromosome:	11:5227002
Gene:	HBB (GeneView)
Functional Consequence:	missense
Allele Origin:	G(germline)/T(germline)/A(germline)/C(germline)
Clinical significance:	Pathogenic
Validated:	by 1000G,by cluster,by frequency,by hapmap
Global MAF:	A=0.0274/137
HGVS:	NC_000011.10:g.5227002T>A, NC_000011.10:g.5227002T>C, NC_000011.10:g.5227002T>G, NC_000011.9:g.5248232T>A, NC_000011.9:g.5248232T>C, NC_000011.9:g.5248232T>G, NG_000007.3:g.70614A>C, NG_000007.3:g.70614A>G, NG_000007.3:g.70614A>T, NM_000518.4:c.20A>C, NM_000518.4:c.20A>G, NM_000518.4:c.20A>T, NP_000509.1:p.Glu7Ala, NP_000509.1:p.Glu7Gly, NP_000509.1:p.Glu7Val

PubMed Varview Protein3D OMIM

rs3168321 has merged into rs334 *[Homo sapiens]*

Please note that the multiple entries that appear should be listed with the latest information at the top of the list. This is the result of multiple rs numbers being assigned to the same SNVs over time. In order to prevent duplication, multiple rs numbers have been merged for most SNVs into a single rs designation.

From this page, the Functional Consequence/type of mutation, missense, is listed, as well as the global or worldwide Mean Allele Frequency (0.0274 or 2.74%), and the effect on the protein. For Sickle Hemoglobin (hemoglobin S) the variant is NM_000518.4(HBB):c.20A>T (p.Glu7Val). Further information that could be sought to include would be MAF in specific populations. For this base position (chromosome 11 at position 5227002), there have been alleles found with all four bases (A/C/G/T), which encode for different amino acids. The missense Glu7Val causes an acidic to non-polar change (at end of this handout are charts for the amino classes).

At the bottom of the information for the rs334 SNV there is a link for OMIM (Online Mendelian Inheritance in Man; An Online Catalog of Human Genes and Genetic Disorders) and for PubMed. PubMed will provide relevant literature citations. Click on the OMIM link and you will get:

+141900 - HEMOGLOBIN--BETA LOCUS; HBB
METHEMOGLOBINEMIA, BETA-GLOBIN TYPE, INCLUDED
Cytogenetic locations: 11p15.4
OMIM: **141900**
Gene summaries   Genetic tests   Medical literature

Click on the main **+141900** - HEMOGLOBIN--BETA LOCUS; HBB link to get a summary of various types of information:

+141900

HEMOGLOBIN--BETA LOCUS; HBB

Other entities represented in this entry:

METHEMOGLOBINEMIA, BETA-GLOBIN TYPE, INCLUDED

ERYTHREMIA, BETA-GLOBIN TYPE, INCLUDED

***HGNC Approved Gene Symbol:* HBB**

***Cytogenetic location:* 11p15.4**   ***Genomic coordinates (GRCh37):* 11:5,246,695-5,248,300** (from NCBI)

Gene-Phenotype Relationships

Location	Phenotype	Phenotype MIM number	Inheritance (in progress)	Phenotype mapping key
11p15.4	Delta-beta thalassemia	141749	AD	3
	Erythremias, beta-			3
	Heinz body anemias, beta-	140700	AD	3
	Hereditary persistence of fetal hemoglobin	141749	AD	3
	Methemoglobinemias, beta-			3
	Sickle cell anemia	603903		3
	Thalassemia-beta, dominant inclusion-body	603902		3
	Thalassemias, beta-	613985		3
	{Malaria, resistance to}	611162		3

Clinical Reasoning Case 1

Nelson College: Table 16

Case History: Breast Cancer and Pacemaker

Classify each of the variants found similar to the Sickle Cell rs334 example provided above. Answer the questions that follow the table of variants. Incorporate this information into your Power Point presentation. In the presentation, you should discuss/review background information concerning the purpose/clinical utility of sequencing, the methodology used, the results from your analysis of the sequencing data, and any conclusions regarding the anomalies/pathologies observed in the cadaver that was selected for sequencing. Specific points that should be addressed in your presentation include DNA preparation and quality, genes associated with the variants, significance of mean allele frequency, advantages and disadvantages of exome sequencing, expected or known biochemical effects of the variants on protein function, associations of the variants and genes with traits and diseases, background information on the associated diseases and traits, and how the variants may have impacted the traits and diseases the cadaver may have experienced and how that may have impacted subsequent health care. Don't forget to list the references and resources used to guide you through this exercise and to prepare your presentation.

**Chromosome**	**Start**	**End**	**Ref**	**Alt**	**dbSNP**	**Depth**	**Zygosity**	**Gene**
chr2	44066247	44066247	G	C	rs11887534	17	Het	ABCG8
chr21	44483184	44483184	A	G	rs5742905	12	Het	CBS
chr1	11856378	11856378	G	A	rs1801133	15	Het	MTHFR
chr1	70904800	70904800	G	T	rs1021737	73	Hom	CTH
chr5	33951693	33951693	C	G	rs16891982	27	Hom	SLC45A2
chr11	68846399	68846399	A	T	rs35264875	28	Het	TPCN2
chr11	88911696	88911696	C	A	rs1042602	73	Het	TYR
chr13	32906729	32906729	A	C	rs144848	122	Hom	BRCA2
chr22	42522613	42522613	G	C	rs1135840	8	Het	NDUFA6-AS1
chr19	41515263	41515263	A	G	rs2279343	14	Het	CYP2B6
chr3	38645420	38645420	T	C	rs1805124	28	Hom	SCN5A

1. Are any of the variants related to susceptibility to breast cancer?
2. Are any of the variants related to susceptibility to a cardiac condition requiring a pacemaker?
3. Are the variants found consistent with the cadaver's skin color?
4. Are any of the variants related to the metabolism of drugs?
5. Have any of the remaining variants been related to any other clinical conditions/disorders?

Clinical Reasoning Case 2

Saunders College: Table 38

Case History: Dementia

Classify each of the variants found similar to the Sickle Cell rs334 example provided above. Answer the questions that follow the table of variants. Incorporate this information into your Power Point presentation. In the presentation, you should discuss/review background information concerning the purpose/clinical utility of sequencing, the methodology used, the results from your analysis of the sequencing data, and any conclusions regarding the anomalies/pathologies observed in the cadaver that was selected for sequencing. Specific points that should be addressed in your presentation include DNA preparation and quality, genes associated with the variants, significance of mean allele frequency, advantages and disadvantages of exome sequencing, expected or known biochemical effects of the variants on protein function, associations of the variants and genes with traits and diseases, background information on the associated diseases and traits, and how the variants may have impacted the traits and diseases the cadaver may have experienced and how that may have impacted subsequent health care. Don't forget to list the references and resources used to guide you through this exercise and to prepare your presentation.

Chromosome	Start	End	Ref	Alt	dbSNP	Depth	Zygosity	Gene
chr20	4680251	4680251	A	G	rs1799990	4	Het	PRNP
chr1	94467548	94467548	C	G	rs41292677	13	Het	ABCA4
chr5	33951693	33951693	C	G	rs16891982	18	Het	SLC45A2
chr5	33963870	33963870	C	T	rs26722	14	Het	SLC45A2
chr11	89017961	89017961	G	A	rs1126809	8	Het	TYR
chr11	68846399	68846399	A	T	rs35264875	34	Het	TPCN2
chr16	89985844	89985844	G	T	rs1805005	47	Hom	MC1R
chr9	132580901	132580901	C	G	rs1801968	14	Het	TOR1A
chr17	72745313	72745313	C	G	rs35910969	9	Het	RAB37

1. Are any of the variants related to susceptibility to dementia?
2. Are the variants found consistent with the cadaver's skin color?
3. Are the variants found consistent with the cadaver's (predicted) hair color?
4. Have any of the remaining variants been related to any other clinical conditions/disorders? If so, which ones?

Clinical Reasoning Case 3

Sherry College: Table 35

Case History: ALS

Classify each of the variants found similar to the Sickle Cell rs334 example provided above. Answer the questions that follow the table of variants. Incorporate this information into your Power Point presentation. In the presentation, you should discuss/review background information concerning the purpose/clinical utility of sequencing, the methodology used, the results from your analysis of the sequencing data, and any conclusions regarding the anomalies/pathologies observed in the cadaver that was selected for sequencing. Specific points that should be addressed in your presentation include DNA preparation and quality, genes associated with the variants, significance of mean allele frequency, advantages and disadvantages of exome sequencing, expected or known biochemical effects of the variants on protein function, associations of the variants and genes with traits and diseases, background information on the associated diseases and traits, and how the variants may have impacted the traits and diseases the cadaver may have experienced and how that may have impacted subsequent health care. Don't forget to list the references and resources used to guide you through this exercise and to prepare your presentation.

Chromosome	Start	End	Ref	Alt	dbSNP	Depth	Zygosity	Gene
chr1	11082635	11082635	A	G	rs80356742	11	Het	TARDBP
chr1	46655645	46655645	C	T	rs74374973	4	Het	POMGNT1
chr8	18258103	18258103	G	A	rs1799930	11	Het	NAT2
chr11	88911696	88911696	C	A	rs1042602	49	Het	TYR
chr11	89017961	89017961	G	A	rs1126809	55	Het	TYR
chr16	89985844	89985844	G	T	rs1805005	48	Het	MC1R
chr5	33951693	33951693	C	G	rs16891982	33	Het	SLC45A2
chr11	68855363	68855363	G	A	rs3829241	6	Het	TPCN2
chr8	21976710	21976710	T	C	rs7014851	17	Het	HR

1. Are any of the variants related to susceptibility to ALS? Could other variants have contributed to the muscle wasting (dystrophy) phenotype?
2. Are the variants found consistent with the cadaver's skin color?
3. Are the variants found consistent with the cadaver's (predicted) hair color?
4. Are any of the variants related to the metabolism of drugs? If so, which ones?
5. Have any of the remaining variants been related to any other clinical conditions/disorders? If so, which ones?

Clinical Reasoning Case 4

Babcock College: Table 3

Case History: gastric cancer

Classify each of the variants found similar to the Sickle Cell rs334 example provided above. Answer the questions that follow the table of variants. Incorporate this information into your Power Point presentation. In the presentation, you should discuss/review background information concerning the purpose/clinical utility of sequencing, the methodology used, the results from your analysis of the sequencing data, and any conclusions regarding the anomalies/pathologies observed in the cadaver that was selected for sequencing. Specific points that should be addressed in your presentation include DNA preparation and quality, genes associated with the variants, significance of mean allele frequency, advantages and disadvantages of exome sequencing, expected or known biochemical effects of the variants on protein function, associations of the variants and genes with traits and diseases, background information on the associated diseases and traits, and how the variants may have impacted the traits and diseases the cadaver may have experienced and how that may have impacted subsequent health care. Don’t forget to list the references and resources used to guide you through this exercise and to prepare your presentation.

chromosome	start	end	ref	alt	dbsnp	depthOfAllele	zygosity	Gene
chr1	55529187	55529187	G	A	rs505151	22	Hom	PCSK9
chr5	33951693	33951693	C	G	rs16891982	27	Hom	SLC45A2
chr16	89986117	89986117	C	T	rs1805007	37	Het	MC1R
chr11	89017961	89017961	G	A	rs1126809	22	Het	TYR
chr17	28576076	28576076	T	C	rs1050565	7	Het	BLMH
chr22	42522613	42522613	G	C	rs1135840	7	Het	CYP2D6
chr5	176520243	176520243	G	A	rs351855	15	Het	FGFR4

1. Are any of the variants related to cancer?
2. Are the variants found consistent with the cadaver’s skin color?
3. Are the variants found consistent with the cadaver’s (predicted) hair color?
4. Are any of the variants related to the metabolism of drugs? If so, which ones?
5. Have any of the remaining variants been related to any other clinical conditions/disorders? If so, which ones?

Clinical Reasoning Case 5

Marks College: Table 26

Case history: Unknown

Classify each of the variants found similar to the Sickle Cell rs334 example provided above. Answer the questions that follow the table of variants. Incorporate this information into your Power Point presentation. In the presentation, you should discuss/review background information concerning the purpose/clinical utility of sequencing, the methodology used, the results from your analysis of the sequencing data, and any conclusions regarding the anomalies/pathologies observed in the cadaver that was selected for sequencing. Specific points that should be addressed in your presentation include DNA preparation and quality, genes associated with the variants, significance of mean allele frequency, advantages and disadvantages of exome sequencing, expected or known biochemical effects of the variants on protein function, associations of the variants and genes with traits and diseases, background information on the associated diseases and traits, and how the variants may have impacted the traits and diseases the cadaver may have experienced and how that may have impacted subsequent health care. Don't forget to list the references and resources used to guide you through this exercise and to prepare your presentation.

chromosome	start	end	ref	alt	dbsnp	depthOfAllele	zygosity	Gene
chr8	16012594	16012594	G	A	rs41341748	33	Het	MSR1
chr6	26093141	26093141	G	A	rs1800562	7	Het	HFE
chr10	115805056	115805056	G	C	rs1801253	29	Hom	ADRB1
chr11	17409572	17409572	T	C	rs5219	25	Het	KCNJ11
chr16	23200963	23200963	G	A	rs5738	23	Het	SCNN1G
chr16	89985844	89985844	G	T	rs1805005	64	Hom	MC1R
chr15	28260053	28260053	G	A	rs1800401	20	Het	OCA2
chr5	33951693	33951693	C	G	rs16891982	18	Het	SLC45A2
chr11	68846399	68846399	A	T	rs35264875	73	Het	TPCN2

1. Are any of the variants related to cancer?
2. Are the variants found consistent with the cadaver's skin color?
3. Are the variants found consistent with the cadaver's (predicted) hair color?
4. Are any of the variants related to the metabolism of drugs? If so, which ones?
5. Have any of the remaining variants been related to any other clinical conditions/disorders? If so, which ones?

Clinical Reasoning Case 6

Durant College: Table 20

Case history: Lung cancer

Classify each of the variants found similar to the Sickle Cell rs334 example provided above. Answer the questions that follow the table of variants. Incorporate this information into your Power Point presentation. In the presentation, you should discuss/review background information concerning the purpose/clinical utility of sequencing, the methodology used, the results from your analysis of the sequencing data, and any conclusions regarding the anomalies/pathologies observed in the cadaver that was selected for sequencing. Specific points that should be addressed in your presentation include DNA preparation and quality, genes associated with the variants, significance of mean allele frequency, advantages and disadvantages of exome sequencing, expected or known biochemical effects of the variants on protein function, associations of the variants and genes with traits and diseases, background information on the associated diseases and traits, and how the variants may have impacted the traits and diseases the cadaver may have experienced and how that may have impacted subsequent health care. Don't forget to list the references and resources used to guide you through this exercise and to prepare your presentation.

chromosome	start	end	ref	alt	dbsnp	depthOfAllele	zygosity	Gene
chr1	26764719	26764719	A	G	rs147394623	5	Het	DHDDS
chr3	45814094	45814094	G	A	rs17279437	10	Het	SLC6A20
chr5	148206885	148206885	C	T	rs1800888	12	Het	ADRB2
chr5	172662014	172662014	G	A	rs28936670	9	Het	NKX2-5
chr10	96741053	96741053	A	C	rs1057910	12	Het	CYP2C9
chr10	43610119	43610119	G	A	rs1799939	45	Het	RET
chr10	88635779	88635779	C	A	rs11528010	15	Het	BMPR1A
chr11	108175462	108175462	G	A	rs1801516	38	Het	ATM

1. Are any of the variants related to cancer?
2. Are the variants found consistent with the cadaver's skin color?
3. Are the variants found consistent with the cadaver's hair color?
4. Are any of the variants related to the metabolism of drugs? If so, which ones?
5. Have any of the remaining variants been related to any other clinical conditions/disorders? If so, which ones?

Clinical Reasoning Case 7

Parkinson College: Table 12
Case history: Myelodysplastic syndrome

Classify each of the variants found similar to the Sickle Cell rs334 example provided above. Answer the questions that follow the table of variants. Incorporate this information into your Power Point presentation. In the presentation, you should discuss/review background information concerning the purpose/clinical utility of sequencing, the methodology used, the results from your analysis of the sequencing data, and any conclusions regarding the anomalies/pathologies observed in the cadaver that was selected for sequencing. Specific points that should be addressed in your presentation include DNA preparation and quality, genes associated with the variants, significance of mean allele frequency, advantages and disadvantages of exome sequencing, expected or known biochemical effects of the variants on protein function, associations of the variants and genes with traits and diseases, background information on the associated diseases and traits, and how the variants may have impacted the traits and diseases the cadaver may have experienced and how that may have impacted subsequent health care. Don't forget to list the references and resources used to guide you through this exercise and to prepare your presentation.

chromosome	start	end	ref	alt	dbsnp	depthOfAllele	zygosity	Gene
chr5	1294166	1294166	C	T	rs61748181	9	Het	TERT
chr17	7579472	7579472	G	C	rs1042522	7	Hom	TP53
chr22	19951271	19951271	G	A	rs4680	14	Het	COMT
chr22	42526694	42526694	G	A	rs1065852	5	Het	CYP2D6
chr7	141672604	141672604	T	C	rs10246939	47	Het	TAS2R38
chr5	33951693	33951693	C	G	rs16891982	32	Hom	SLC45A2
chr11	89017961	89017961	G	A	rs1126809	9	Het	TYR
chr16	89986117	89986117	C	T	rs1805007	16	Het	MC1R
chr12	6143978	6143978	C	T	rs41276738	11	Het	VWF
chr1	169519049	169519049	T	C	rs6025	28	Hom	F5

1. Are any of the variants related to cancer?
2. Are the variants found consistent with the cadaver's skin color?
3. Are the variants found consistent with the cadaver's hair color?
4. Are any of the variants related to the metabolism of drugs? If so, which ones?
5. Have any of the remaining variants been related to any other clinical conditions/disorders? If so, which ones?

Supplementary Figure 1. Agarose gel electrophoresis of cadaver DNAs subject to PCR amplification of a 1069 bp fragment of the CPB gene. Quick-load 1 kb DNA Ladder (New England BioLabs). Lane 1: Heart DNA corresponding to Figure 1 lane 2. Lane 2: Heart DNA corresponding to Figure 2 lane 2. Lane 3: Heart DNA corresponding to Figure 2 lane 4. Lane 4: Liver DNA corresponding to Figure 1 lane 1. Lane 5: Skeletal Muscle DNA corresponding to Figure Figure 2 lane 5: Skin DNA corresponding to Figure 2 lane 6. Lane 7: Liver DNA corresponding to Figure 1 lane 3.

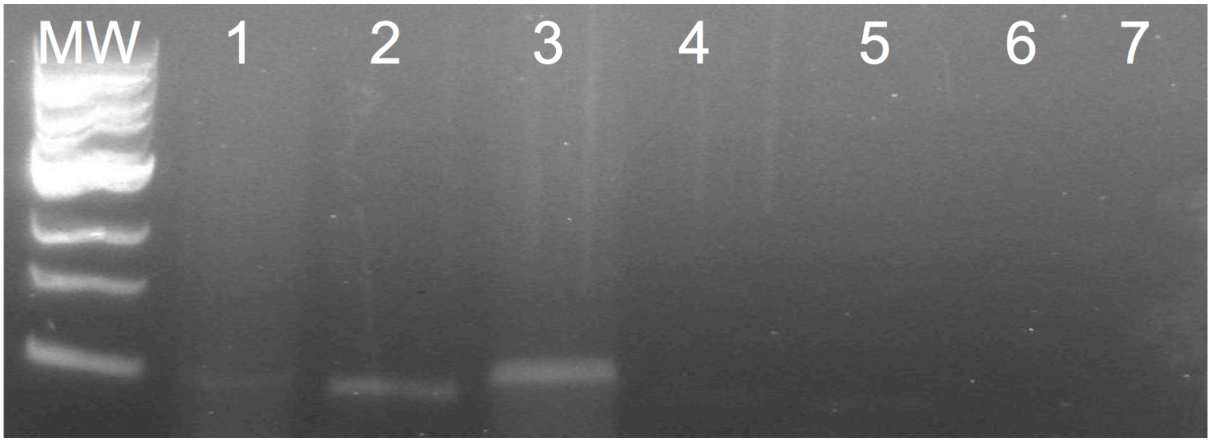

Supplementary Table 1. Amount of basepairs and reads generated by exome sequencing of cadaver DNA.

Sample	Basepairs	Reads
1	4,209,773,904	33,410,904
2	3,469,129,272	27,532,772
3	3,960,508,104	31,432,604
4	5,053,853,952	40,109,952
5	4,863,811,680	38,601,680
6	4,479,061,356	35,548,106
7	3,793,449,996	30,106,746

FastQC Report

Thu 8 Oct 2015
index21_GTTTCG_L001-L002_R2_001.fastq.gz

Summary

- Basic Statistics
- Per base sequence quality
- Per sequence quality scores
- Per base sequence content
- Per base GC content
- Per sequence GC content
- Per base N content
- Sequence Length Distribution
- Sequence Duplication Levels
- Overrepresented sequences
- Kmer Content

Basic Statistics

Measure	Value
Filename	index21_GTTTCG_L001-L002_R2_001.fastq.gz
File type	Conventional base calls
Encoding	Sanger / Illumina 1.9
Total Sequences	16705452
Filtered Sequences	0
Sequence length	126
%GC	47

Per base sequence quality

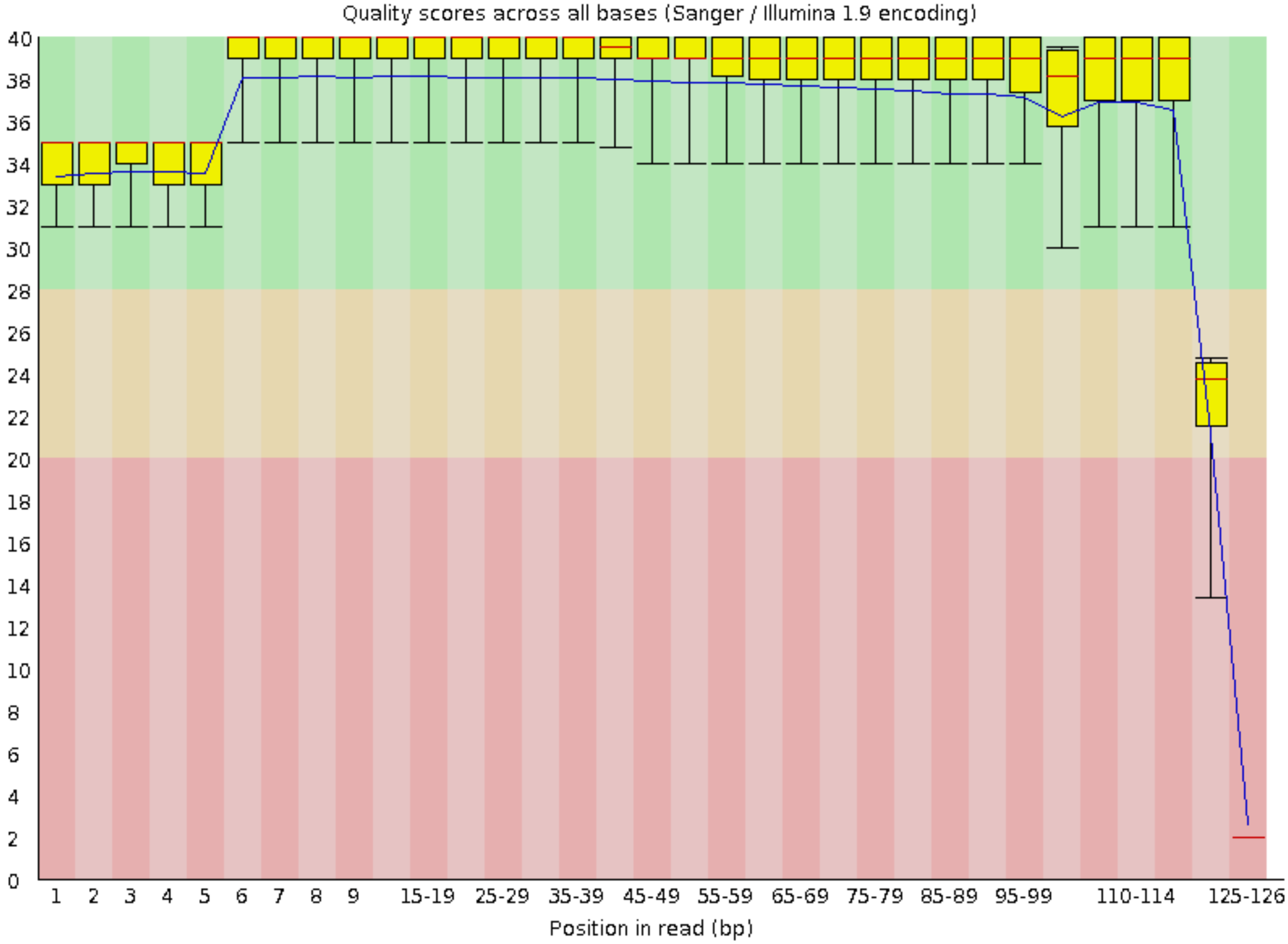

Per sequence quality scores

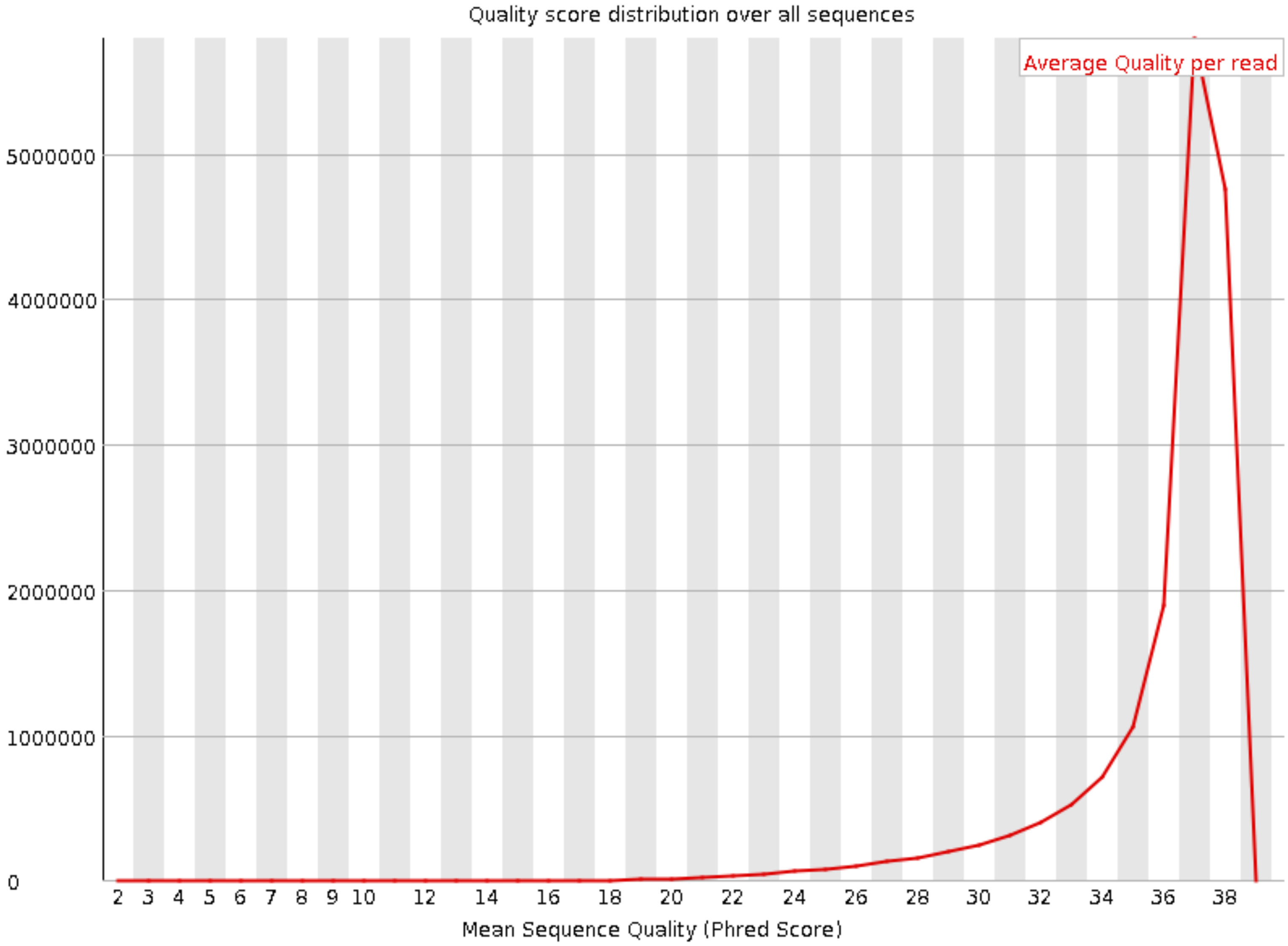

Per base sequence content

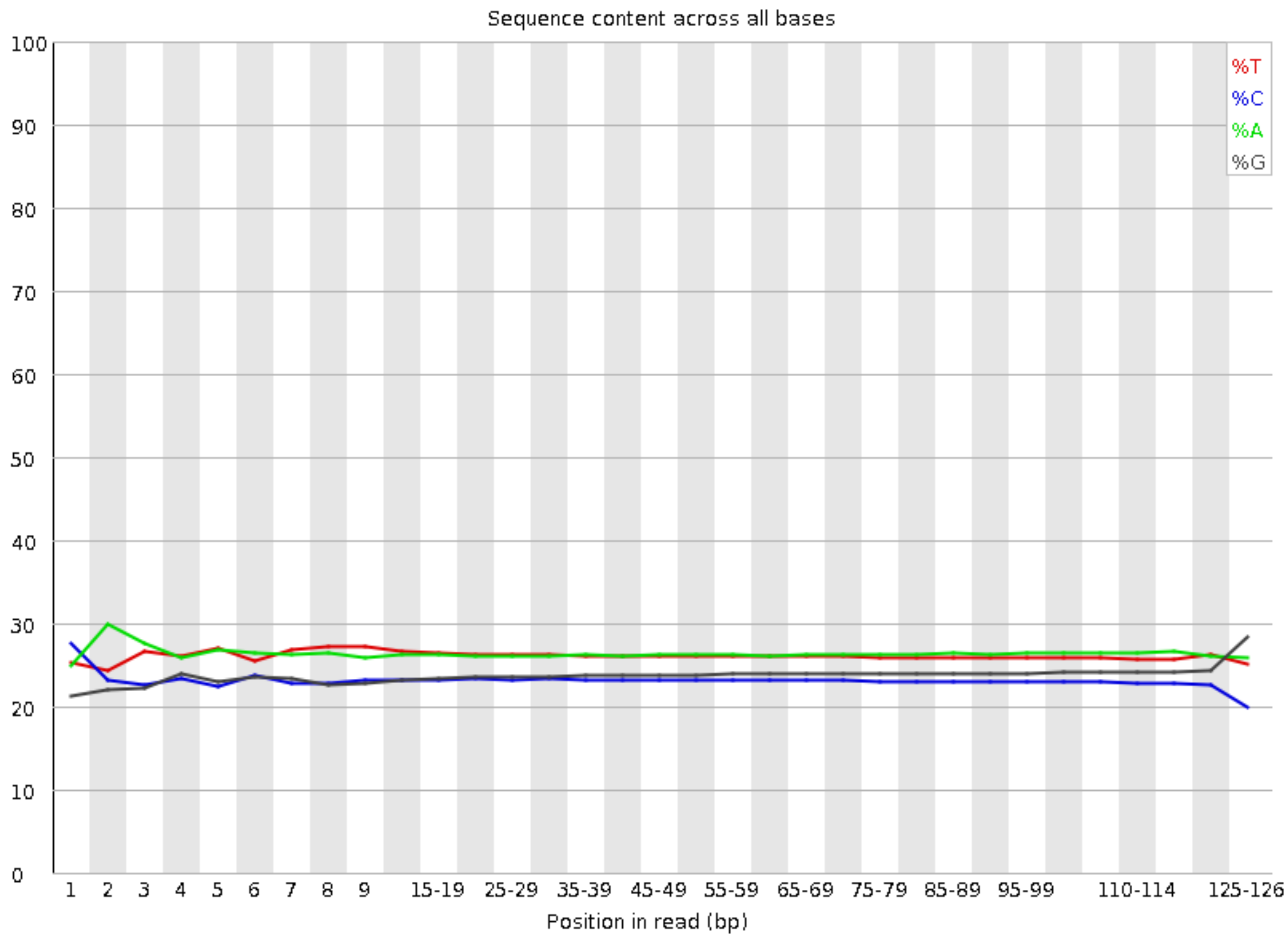

Per base GC content

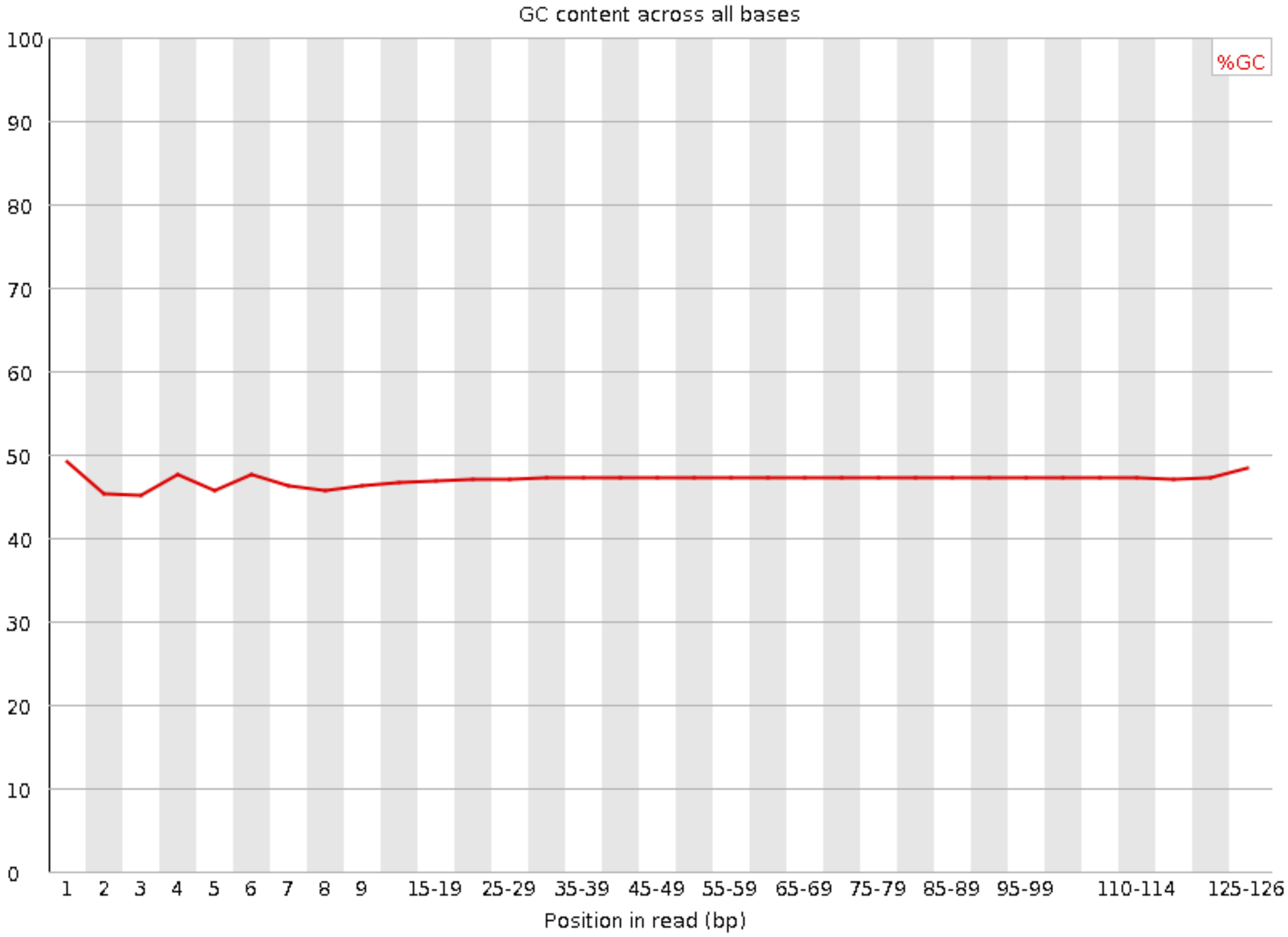

Per sequence GC content

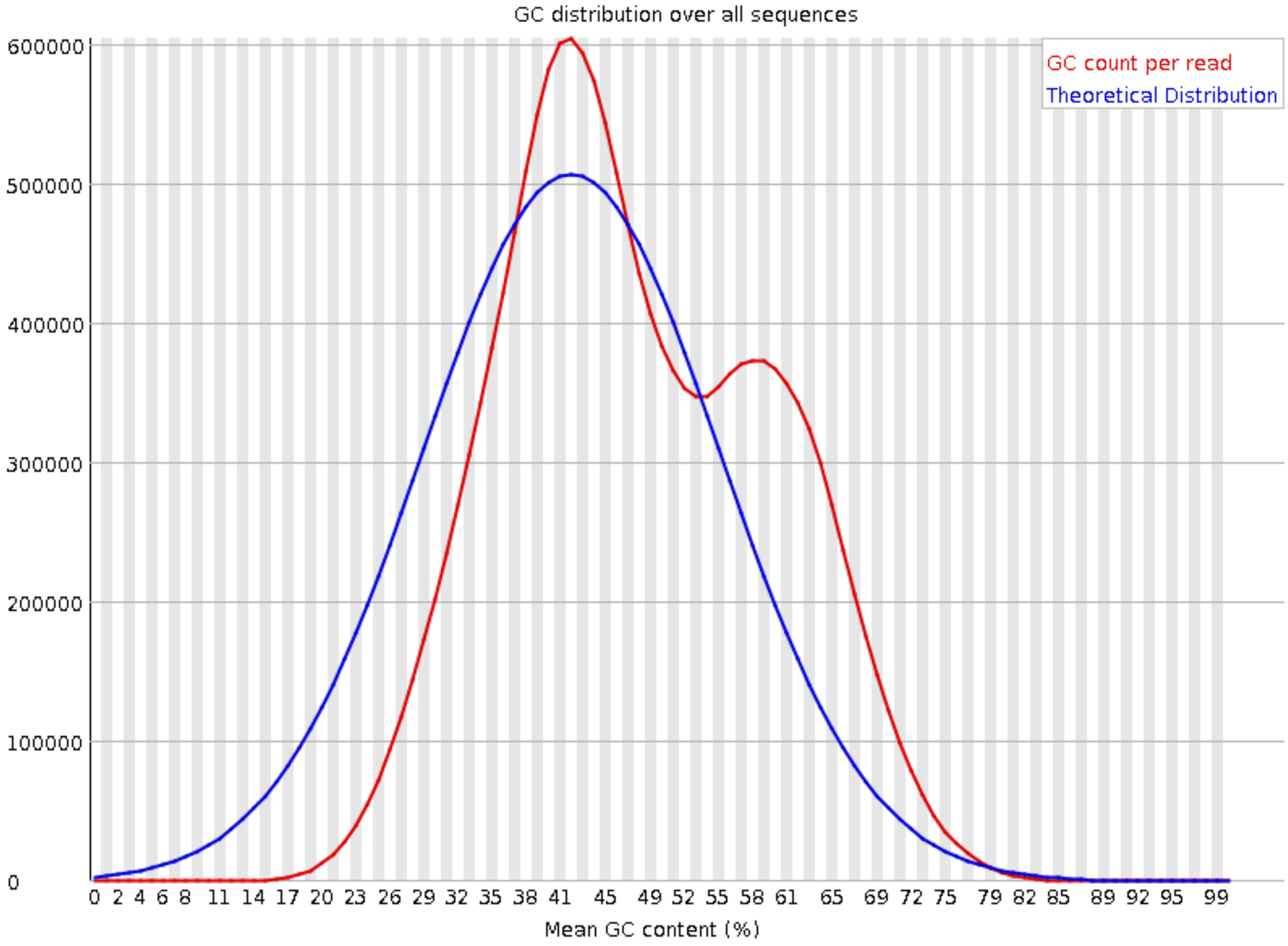

Per base N content

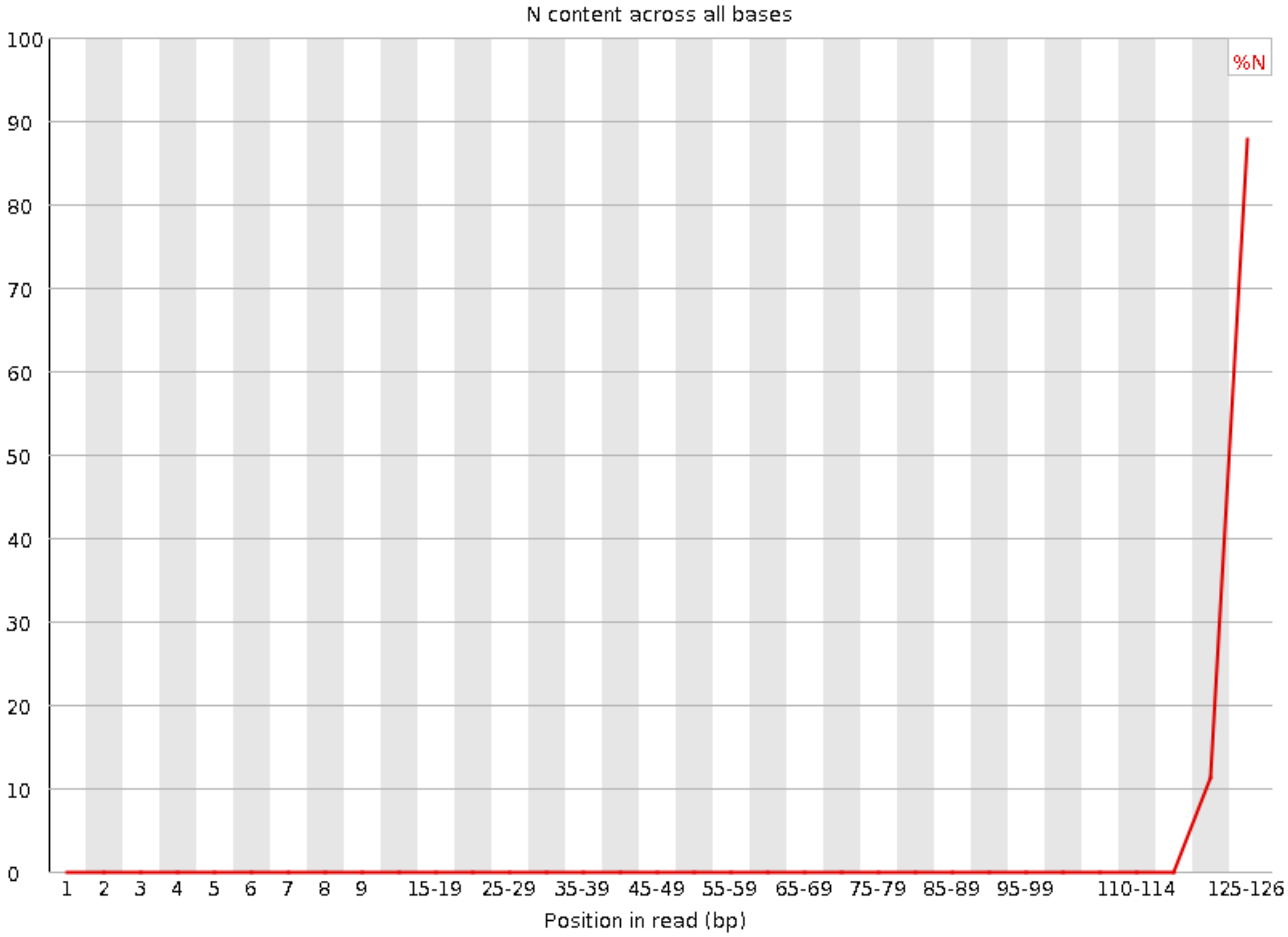

Sequence Length Distribution

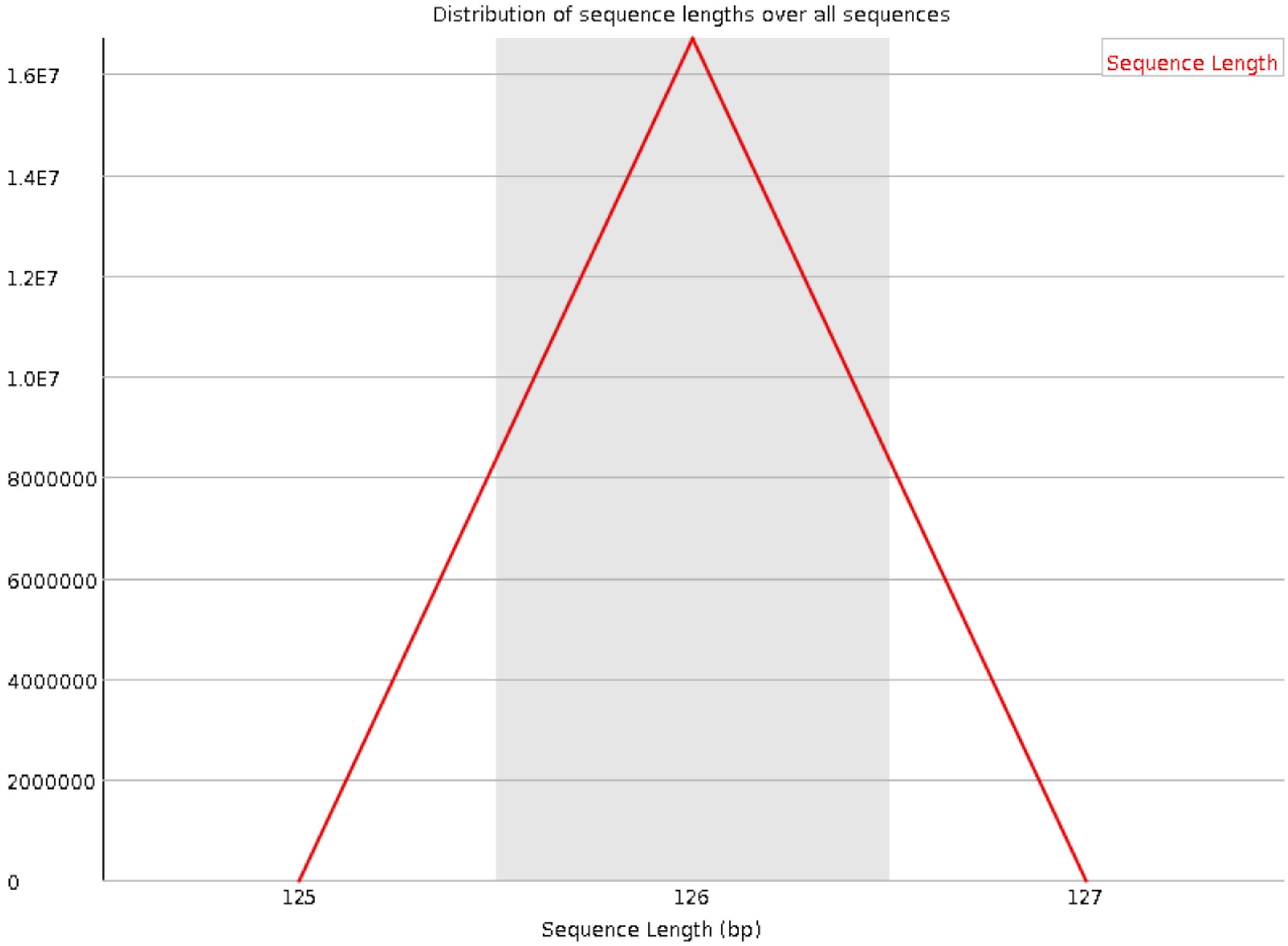

Sequence Duplication Levels

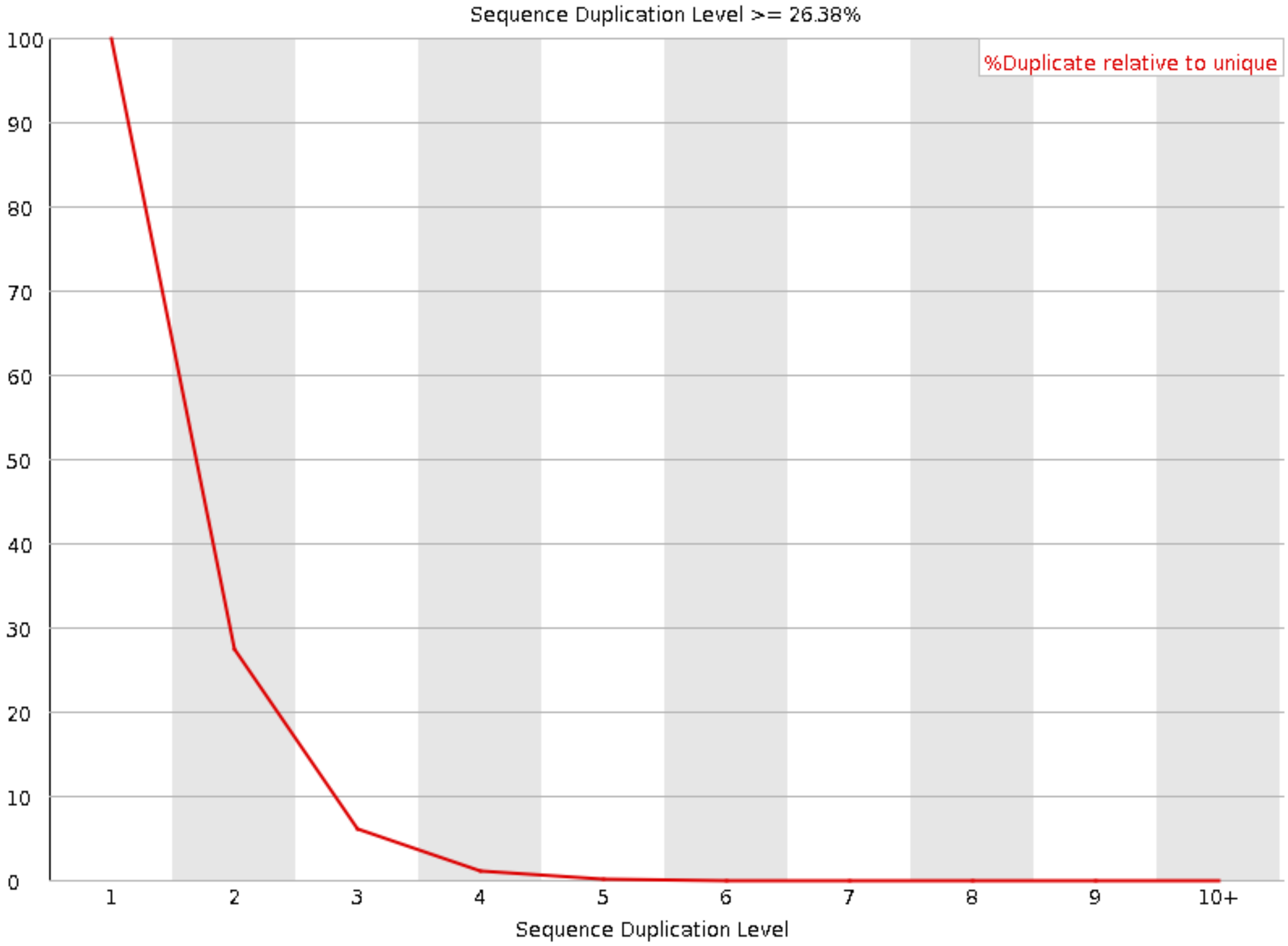

Overrepresented sequences

No overrepresented sequences

Kmer Content

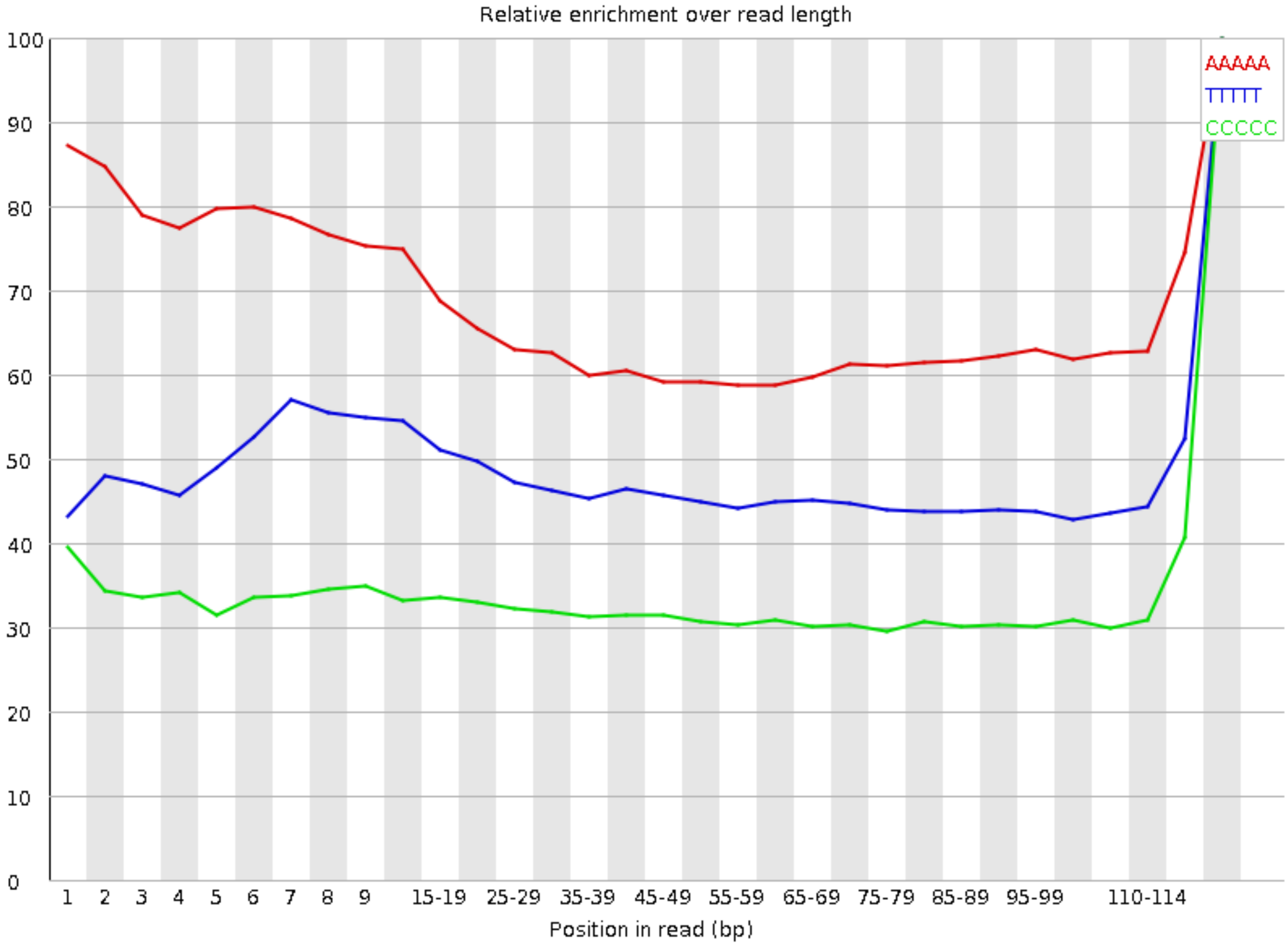

Sequence	Count	Obs/Exp Overall	Obs/Exp Max	Max Obs/Exp Position
AAAAA	7830240	3.0142257	4.673934	120-122
TTTTT	7065535	2.8428354	6.0777855	120-122
CCCCC	2390125	1.745329	5.4111013	120-122

Produced by FastQC (version 0.10.1)

Cadaver [redacted]

Exome Sequencing Project

82 yo Female - Cause of Death

NCBI

dbSNP
Short Genetic Variations

dbVar | ClinVar | GaP | PubMed | Nucleotide | Protein

Search small variations in dbSNP or large structural variations in dbVar

Search Entrez dbSNP for Go

Have a question about dbSNP? Try searching the SNP FAQ Archive! Go

GENERAL
RSS Feed
Contact Us
Site Map
dbSNP Homepage
NCBI Variation
Resources
Announcements
dbSNP Summary
FTP Download
HUMAN VARIATION
SNP SUBMISSION
DOCUMENTATION
SEARCH
RELATED SITES

Reference SNP (refSNP) Cluster Report: rs1805007 ** other **

RefSNP	
**Organism:**	human (Homo sapiens)
**Molecule Type:**	Genomic
**Created/Updated in build:**	89/144
**Map to Genome Build:**	107/Weight
**Validation Status:**	
**Citation:**	PubMed
**Association:**	NHGRI GWAS PheGenI

Allele	
**Variation Class:**	SNV: single nucleotide variation
**RefSNP Alleles:**	C/G/T (FWD)
**Allele Origin:**	C:germline T:germline
**Ancestral Allele:**	C
**Variation Viewer:**	VarView OMIM
**Clinical Significance:**	other
**MAF/MinorAlleleCount:**	T=0.0186/93
**MAF Source:**	1000 Genomes

HGVS Names
NC_000016.10:g.89919709C>T
NC_000016.9:g.89986117C>T
NG_012026.1:g.6831C>T
NG_027810.1:g.2701C>T
NM_002386.3:c.451C>T
NP_002377.4:p.Arg151Cys

Links , Linkout

SNP Details are organized in the following sections:
GeneView | Map | Submission | Fasta | Resource | Diversity | Validation

Integrated Maps (Hint: click on 'Chr Pos' to see variant in the new NCBI variation viewer)

GeneView

Submitter records for this RefSNP Cluster

The submission **ss40730574** has the longest flanking sequence of all cluster members and was used to instantiate sequence for **rs1805007** during BLAST analysis for the current build.

NCBI Assay ID	Handle|Submitter ID	Validation Status	ss to rs Orientation /Strand	Alleles	5' Near Seq 30 bp	3' Near Seq 30 bp	Entry Date	Update Date	Build Added	[illegible]
ss2425920	HGBASE|SNP000062986		fwd/B	C/T	ctacatctccatcttctacgcactg	gctaccacagcatcgtgaccctgcc	11/07/00	10/10/03	89	[illegible]

82 yo Female

Skin Color: Caucasian

Eye Color: Blue

Hair Color: Grey/Some black

COD: Myelodysplastic Syndrome

- **Insufficient production of all blood cells.**
- **What properties are unique to blood cell differentiation?**

Aspect of Hematopoiesis Relevant to MDS

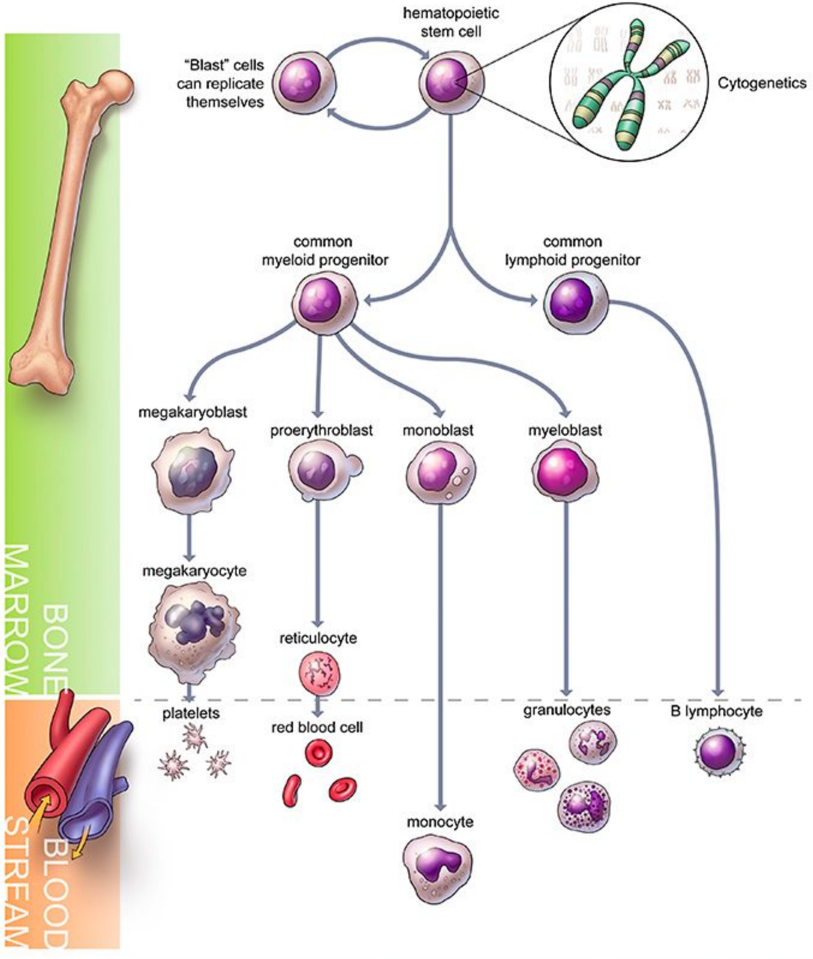

Visual Art: © 2013 The University of Texas MD Anderson Cancer Center

Tyrosinase: *G->C* Arg402Gln

Normal Gene Function: melanin synthesis

SNV rs1126809 - Heterozygous

- 8.13% MAF
- mutated tyrosinase is thermolabile and subject to ER retention
- implicated in squamous cell carcinoma and melanoma
- when compounded with other heterozygous mutations associated with type 1 oculocutaneous albinism

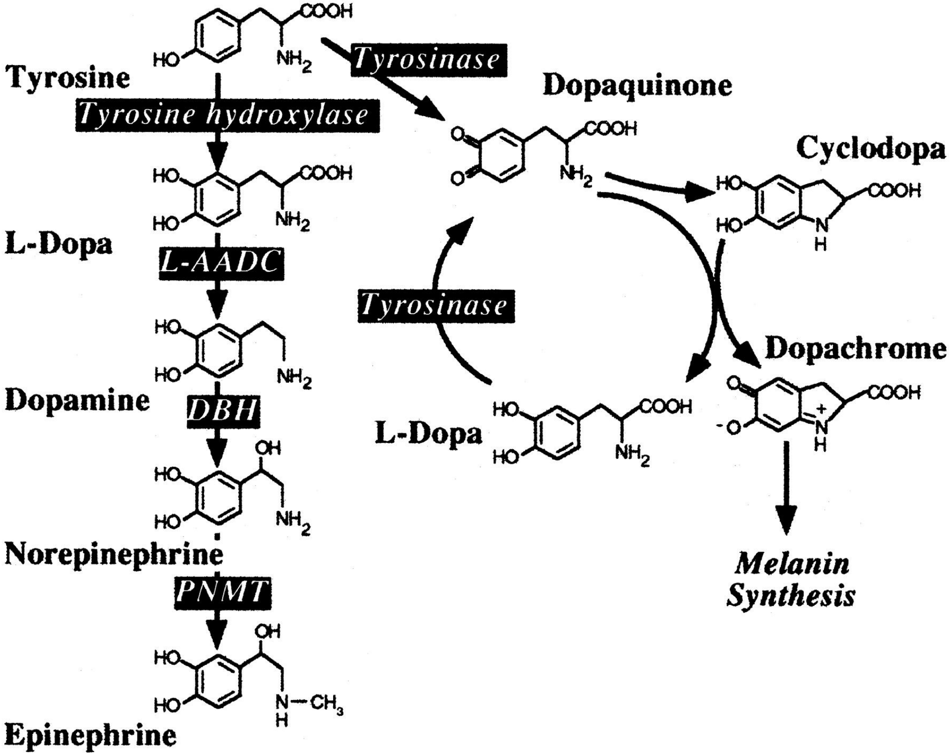

Melanocortin 1 Receptor: *C->T* Arg151Cys

Normal Gene Function: ratio of eumelanin to pheomelanin

SNV rs1805007 - Heterozygous

- 1.86% MAF
- strong association with red hair, light skin
- In conjunction with other mutations or complete LOF cause elevated risk of melanoma

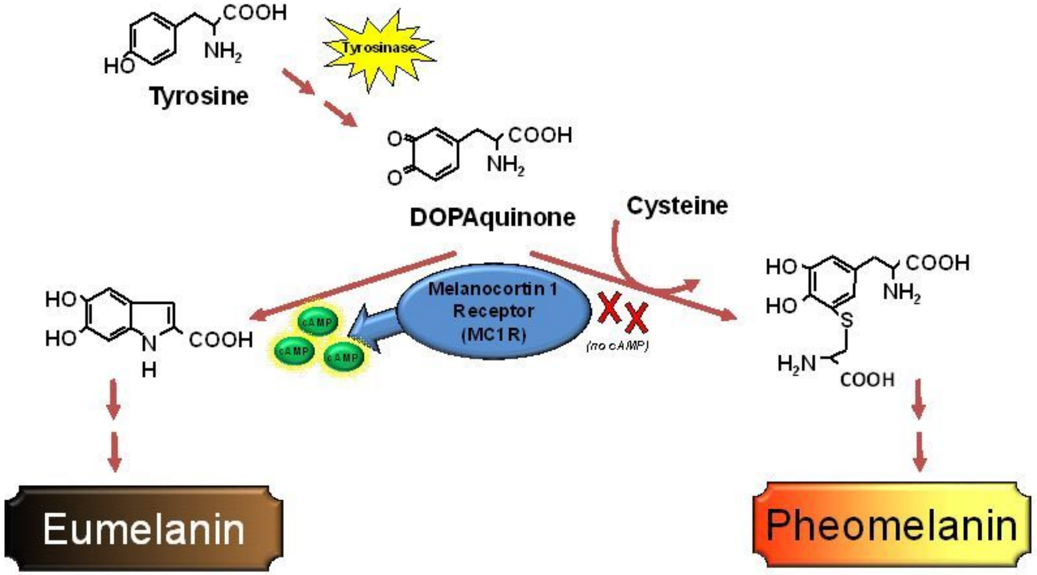

TASR38 Taste Receptor, Type II: *A-->G* Missense: Ile-->Val

Normal Gene Function: **encodes a seven-transmembrane G protein-coupled receptor that controls the ability to taste glucosinolates, a family of bitter-tasting compounds**

SNV rs10246939

- 47.94% MAF
- SNP not associated with any increased risk of cancer, hair, skin, or eye color
- May be responsible for having the ability to taste or not taste bitter compounds
- Patient Heterozygous

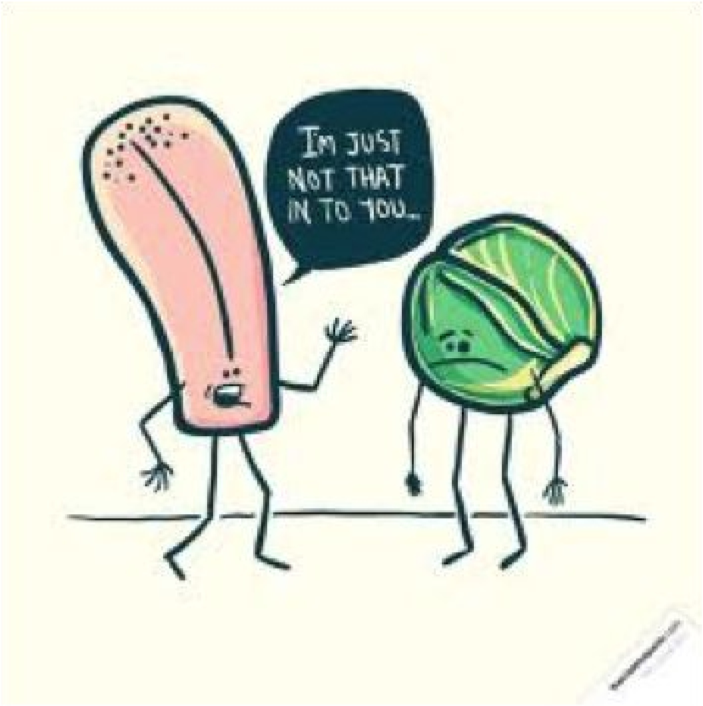

SLC45A2 protein: *C-->G* Missense: Phe-->Leu

Normal Gene Function: Unknown, but likely melanin synthesis

SNV rs16891982

- 0.2750 (27.50%) MAF
- SNP associated with increased risk of basal cell carcinoma and decreased risk of melanoma
- Commonly found in compound heterozygous state in caucasian individuals with dark hair and eye color **(SHEP5; 227240) , Graf et al. (2005))**
- Patient Here was homozygous but had same features

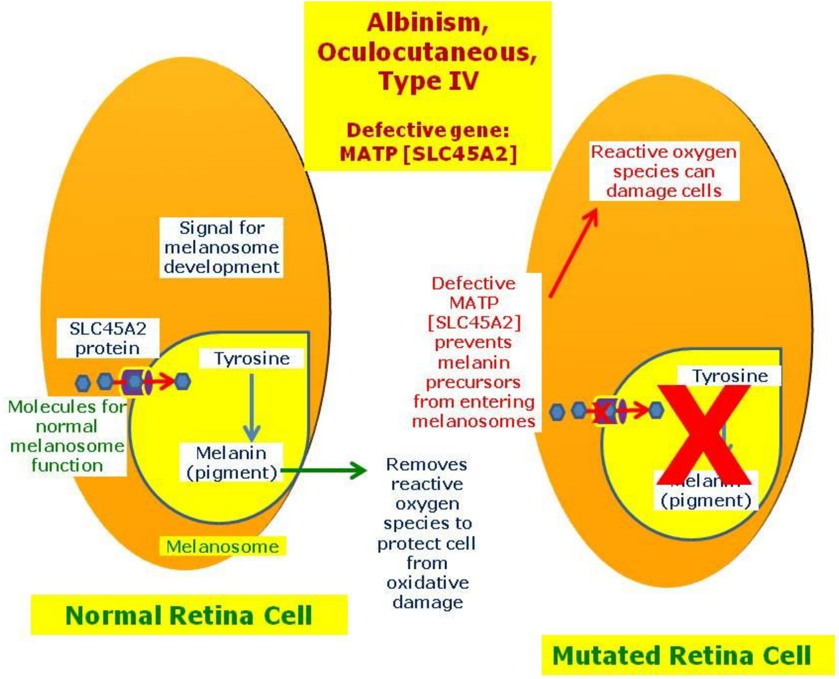

p53: the protein that does (seemingly) everything

Science’s “molecule of the year” in 1993

- More information about p53 than any one person could reasonably digest
- 4,500 references by 1995, >78k results on Pubmed today
- lots of functions already known with more to be discovered

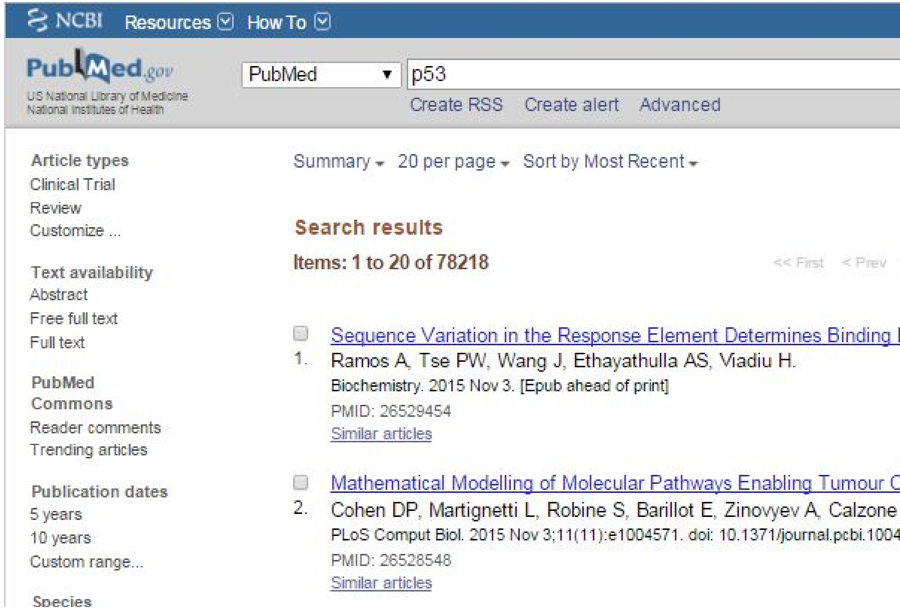

p53 mutation rs1042522

- Pro72Arg SN**P** located in a proline rich domain, allele frequency ~46%
- Pro72Arg polymorphism is **better** at inducing apoptosis (6)
    - ......under some conditions that aren't w[illegible] defined
- Arg/Arg homozygotes are 7x more likely vs. heterozygotes for HPV related tumorigenesis (5)
- Arg/Arg homozygotes were ~2x less likely to get colorectal cancer vs. Arg/Pro heterozygoes (pro/pro homozygotes not studied, ref 7) and got
- Arg/Arg homozygotes got colon cancer later vs. heterozygotes and Pro/Pro homozygotes(8)
- different alleles interact differently with MDM2 variants (a p53 repressor), get lots of possible outcomes (9)

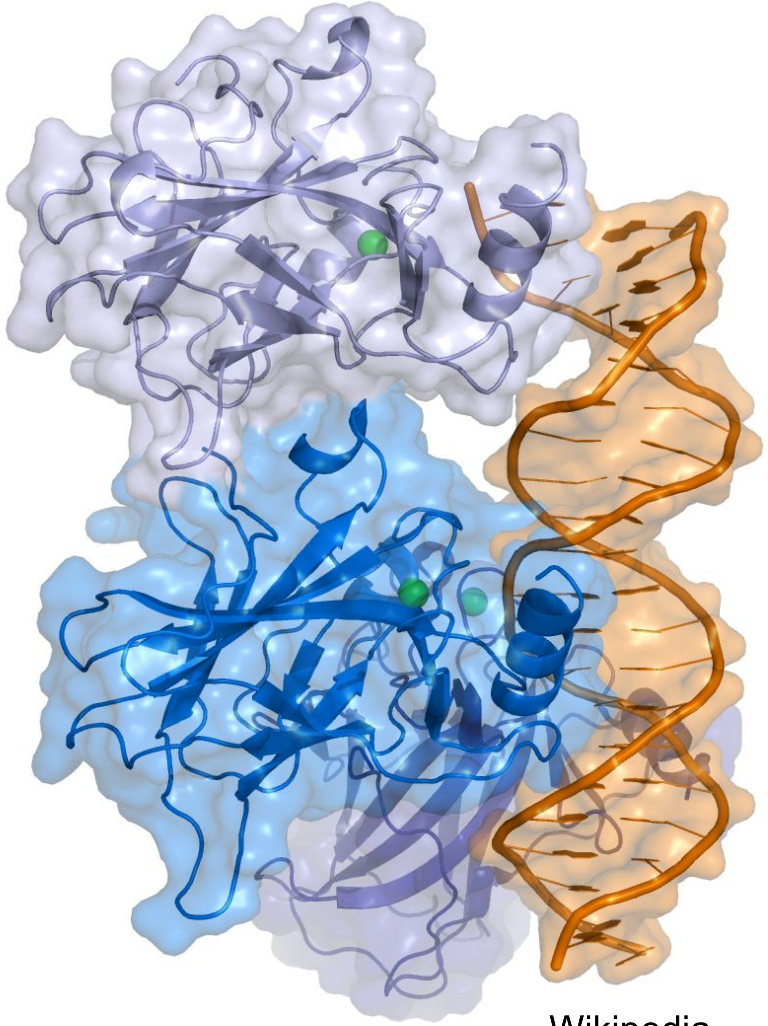

Wikipedia

There’s more! rs1042522, cont.

- Arg72 version binds less tightly to IASPP (most evolutionarily conserved p53 inhibitor) than Pro72 version (10)
- in patients with a germline mutation in MMR, arg homozygotes develop cancer later than hets and pro/pro (13)

Bottom line:

- better survival in Pro72 allele
    - Life expectancy pro/pro > pro/arg > arg/arg in a 12 year study (11)

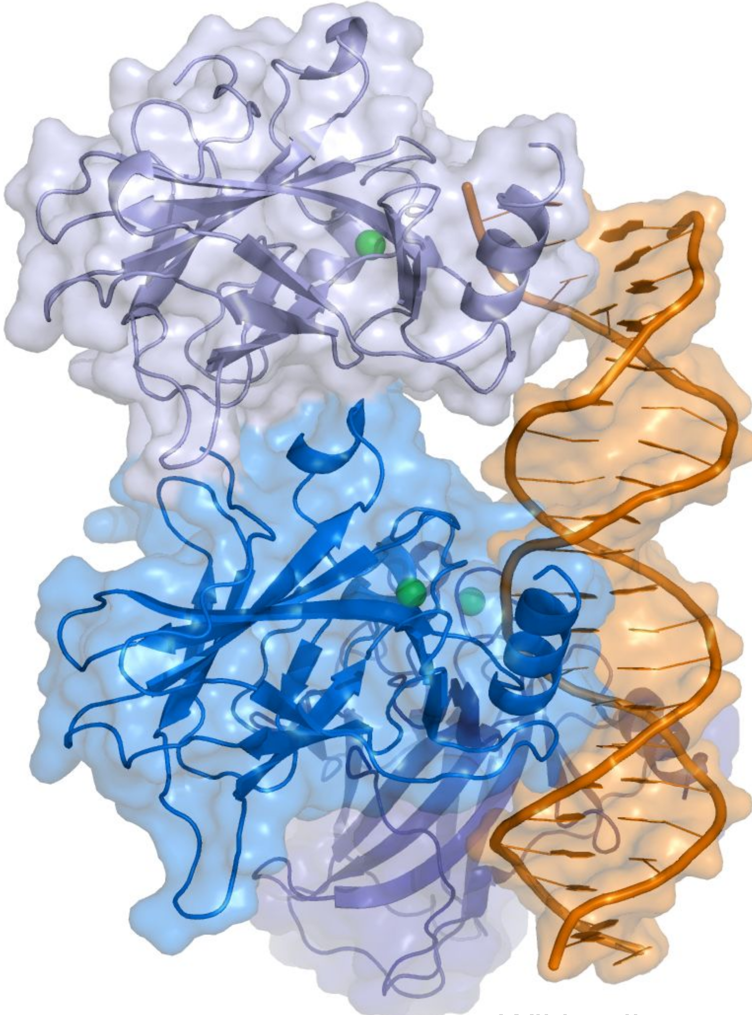

Wikipedia

Actually, there's even more...

Since Pro72Arg didn't have enough cancer related phenotypes...

- Pro72Arg mutation is **protective** in rate of lung function decline in smokers (12)
- **Different** p53 mutations regulate the production of some metabolism intermediates and have been implicated in hair loss.

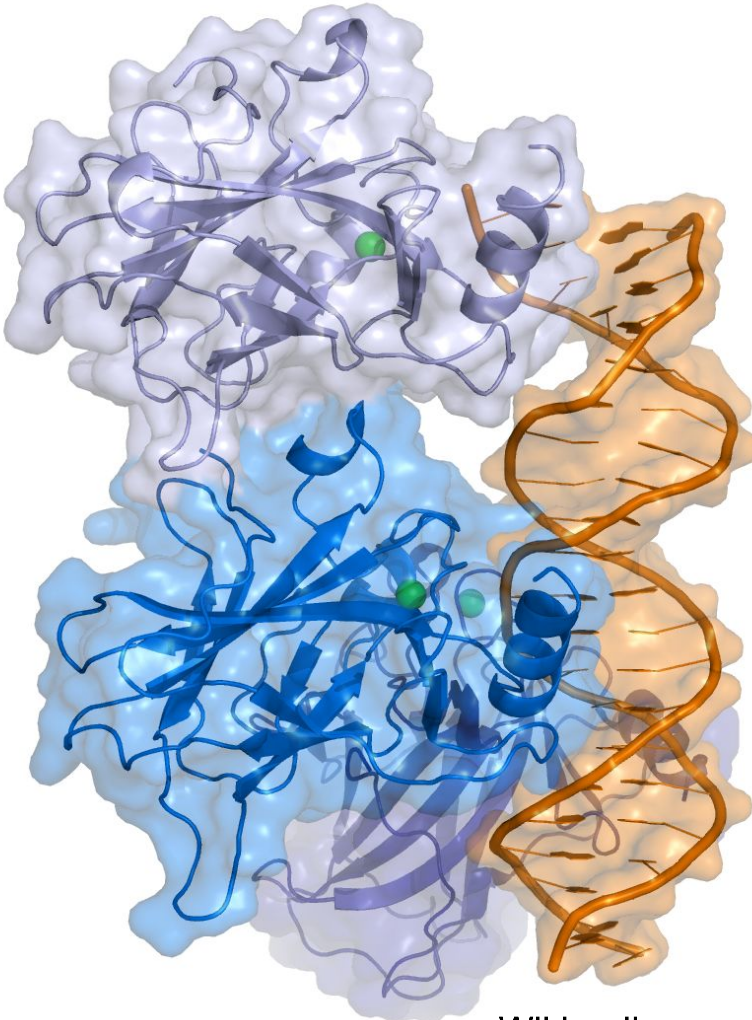

Wikipedia

TERT: Telomerase Reverse Transcriptase

- TERT is the catalytic subunit of telomerase
- rs61748181, heterozygote SNV in the TERT coding sequence C>T
- Global Mean Allele Frequency: 0.96%
- Implicated in being part of hereditary Aplastic Anemia
    - But complex interaction with other mutations (IFN-gamma and other) that’s not well described

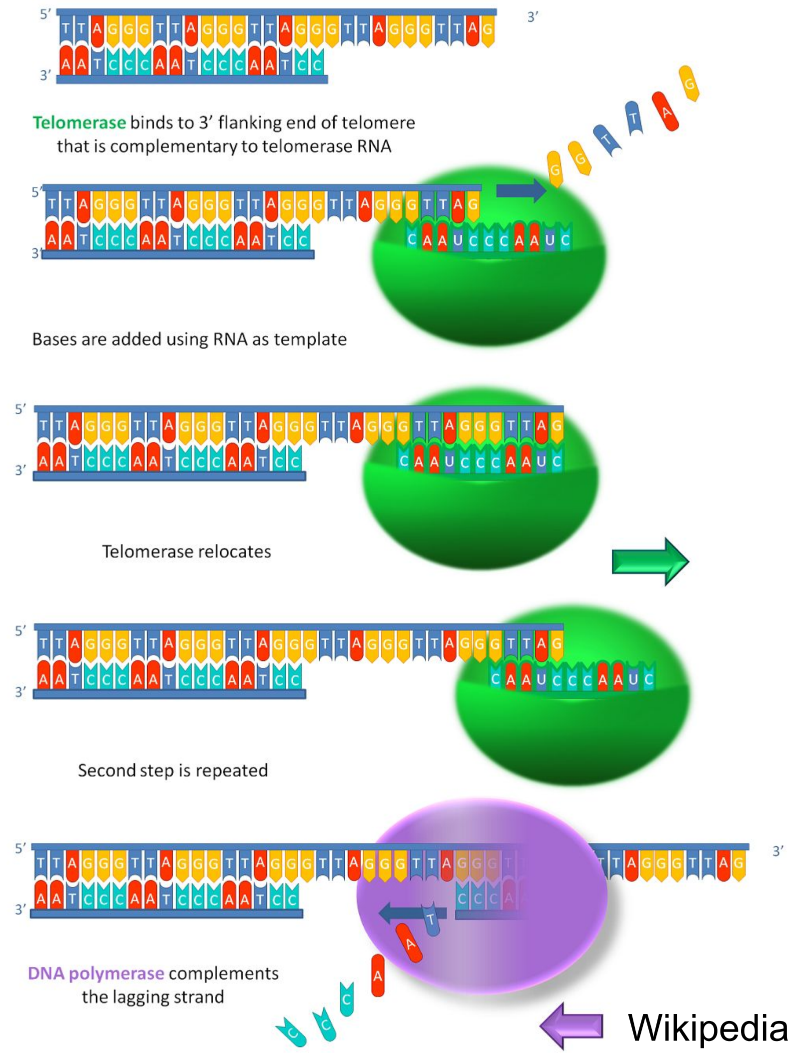

Wikipedia

Von Willebrand factor: C → T, missense: Arg → Gln

- Normal gene Function: adhesion of platelets to the site of vascular injury and transport of thrombotic protein factor VIII

SNV, **rs41276738**

- 0018 (.18%) MAF
- SNV is not associated with cancer or any particular skin or hair color.
- Mutations (including this SNV) are associated with Von Willebrand Disease, a common and complex hereditary bleeding disorder

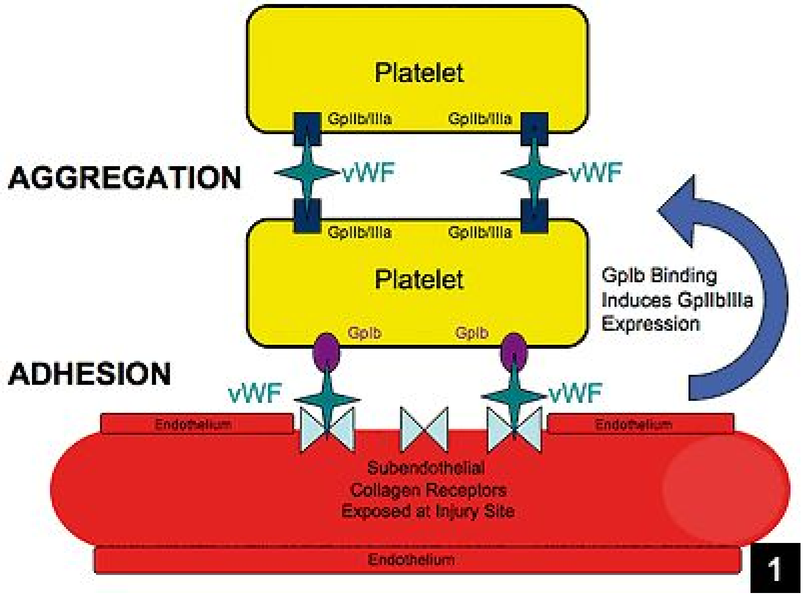

Catechol-O-methyltransferase (COMT)

Normal Gene Function: metabolic degradation of catecholamines (dopamine, epi, norepi)

SNV rs4680:

missense mutation from G → A, switches Valine to Methionine at #158

Global mean allele frequency is 0.3692 (36.92%)

The Met form of COMT is less enzymatically active than the Val form

Puts patients at higher risk of developing schizophrenia and OCD

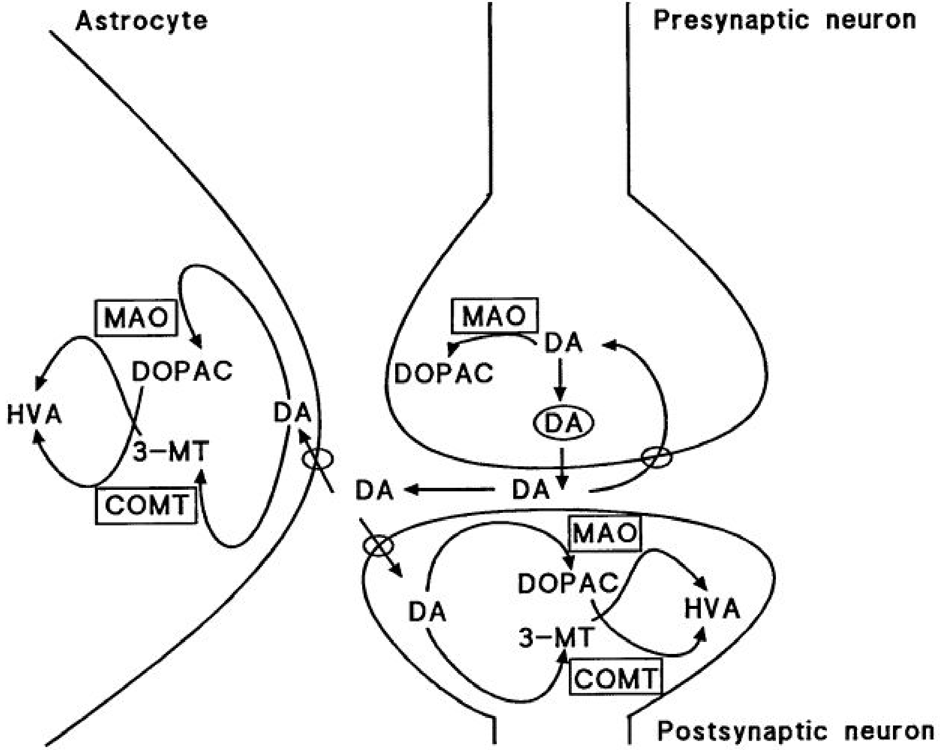

Gene CYP2D6

Involved in the hepatic cytochrome P450 system → responsible for metabolism and elimination of many endogenous and exogenous substances

Gene encodes for Cytochrome P450 2D6

SNV rs1065852:

Global MAF is 0.2380 (23.80%)

missense mutation C → T

Results in poor drug metabolism, esp. that of Debrisoquine (antihypertensive)

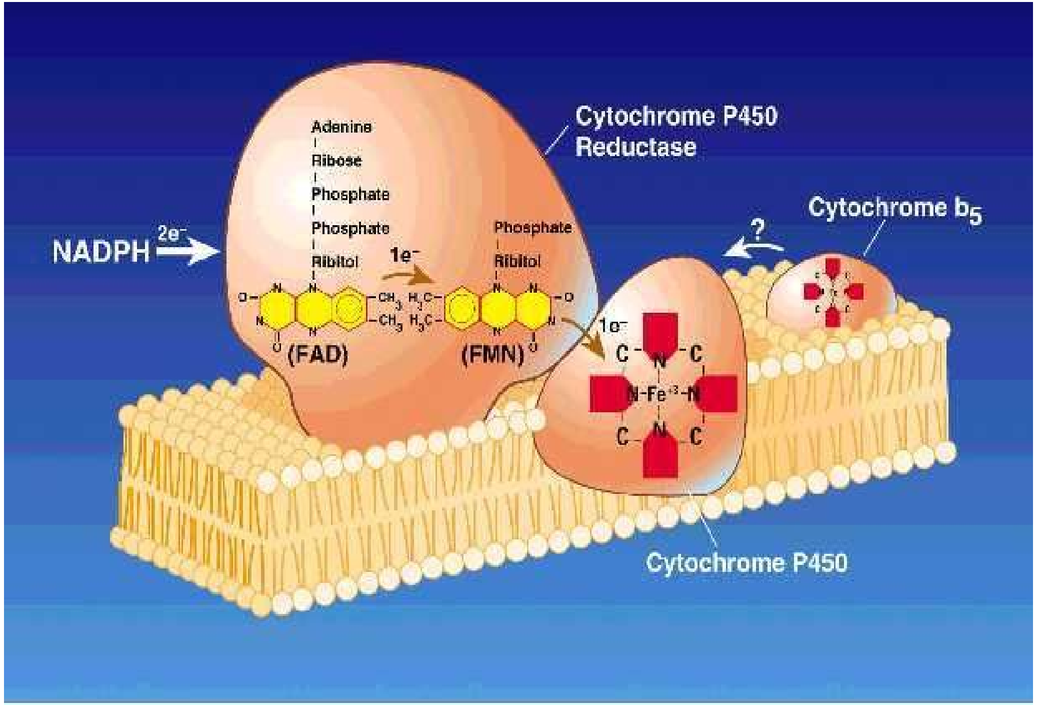

Factor V

-rs6025 encodes a missense mutation on the factor V gene

MAF=0.0060 (0.6%)

-Switches G to A at position 1746 (R534Q)

-Produces factor V Leiden, which cannot be broken down by the anticoagulant activated protein C

-Leads to hypercoagulability, highly associated with DVT and pulmonary embolism

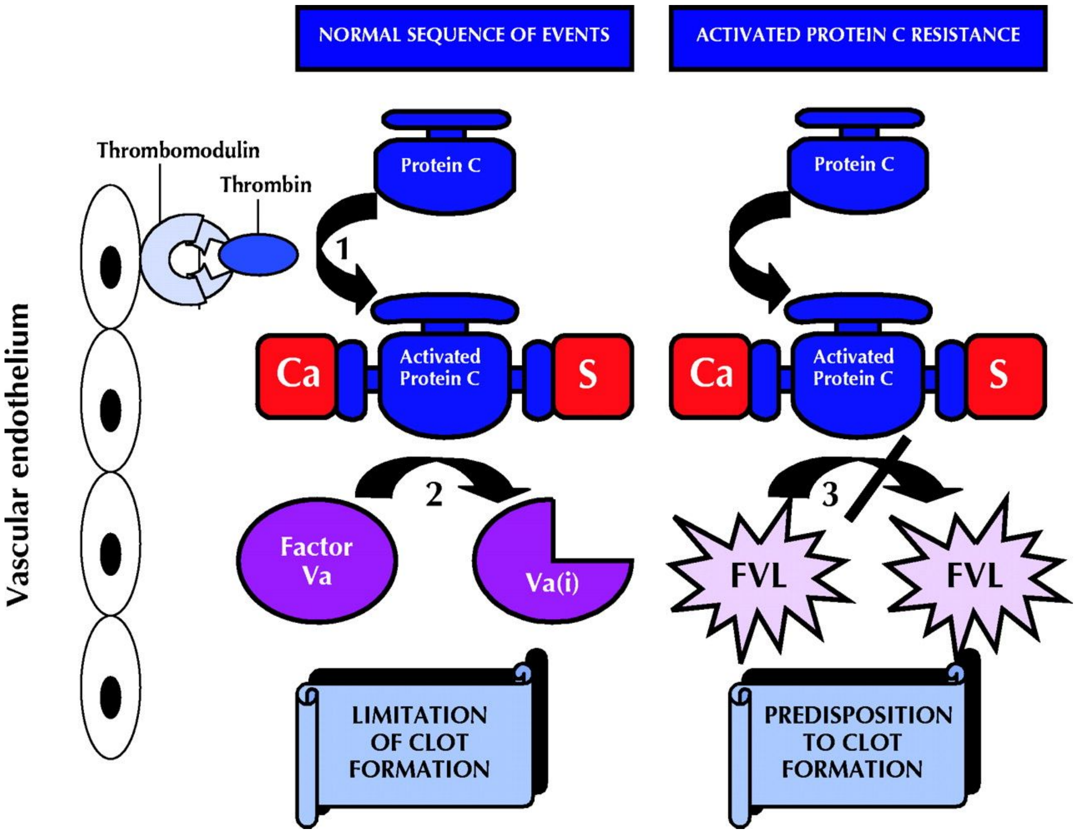

Conclusion

Reminder: COD was Myelodysplastic Syndrome - an insufficient supply of blood cells

What mutations are likely to impact this?

Mutation in vWF results in increased bleeding, this could have contributed to the patient's anemia

TERT: problems in telomerases preferentially cause problems in high turn over cells...like blood products.

References

1: Levine, A. J., Momand, J., Finlay, C. A. The p53 tumour suppressor gene. Nature 351: 453-456, 1991. [PubMed: 2046748] [Full Text]
2: Levine, A. J. p53, the cellular gatekeeper for growth and division. Cell 88: 323-331, 1997. [PubMed: 9039259] [Full Text]
3. Sulem, P., Gudbjartsson, D. F., Stacey, S. N., Helgason, A., Rafnar, T., Magnusson, K. P., Manolescu, A., Karason, A., Palsson, A., Thorleifsson, G., Jakobsdottir, M., Steinberg, S., and 13 others. Genetic determinants of hair, eye and skin pigmentation in Europeans.Nature Genet. 39: 1443-1452, 2007.
4. Valverde, P., Healy, E., Jackson, I., Rees, J. L., Thody, A. J. Variants of the melanocyte-stimulating hormone receptor gene are associated with red hair and fair skin in humans. Nature Genet. 11: 328-330, 1995.
5. Storey, A., Thomas, M., Kalita, A., Harwood, C., Gardiol, D., Mantovani, F., Breuer, J., Leigh, I. M., Matlashewski, G., Banks, L. Role of a p53 polymorphism in the development of human papilloma-virus-associated cancer. Nature 393: 229-234, 1998. [PubMed: 9607760] [Full Text]
6. Dumont, P., Leu, J. I.-J., Pietra, A. C. D., III, George, D. L., Murphy, M. The codon 72 polymorphic variants of p53 have markedly different apoptotic potential. Nature Genet. 33: 357-365, 2003. [PubMed: 12567188] [Full Text]
7. Jones, J. S., Chi, X., Gu, X., Lynch, P. M., Amos, C. I., Frazier, M. L. p53 polymorphism and age of onset of hereditary nonpolyposis colorectal cancer in a Caucasian population. Clin. Cancer Res. 10: 5845-5849, 2004. [PubMed: 15355915] [Full Text]
8. Kruger, S., Bier, A., Engel, C., Mangold, E., Pagenstecher, C., von Knebel Doeberitz, M., Holinski-Feder, E., Moeslein, G., Schulmann, K., Plaschke, J., Ruschoff, J., Schackert, H. K., German HNPCC Consortium. The p53 codon 72 variation is associated with the age of onset of hereditary non-polyposis colorectal cancer (HNPCC). J. Med. Genet. 42: 769-773, 2005. [PubMed: 16199549] [Full Text]
9. Bougeard, G., Baert-Desurmont, S., Tournier, I., Vasseur, S., Martin, C., Brugieres, L., Chompret, A., Bressac-de Paillerets, B., Stoppa-Lyonnet, D., Bonaiti-Pellie, C., Frebourg, T. Impact of the MDM2 SNP309 and p53 arg72-to-pro polymorphism on age of tumour onset in Li-Fraumeni syndrome. (Letter) J. Med. Genet. 43: 531-533, 2006. [PubMed: 16258005] [Full Text]
10. Bergamaschi, D., Samuels, Y., Sullivan, A., Zvelebil, M., Breyssens, H., Bisso, A., Del Sal, G., Syed, N., Smith, P., Gasco, M., Crook, T., Lu, X. iASPP preferentially binds p53 proline-rich region and modulates apoptotic function of codon 72- polymorphic p53. Nature Genet. 38: 1133-1141, 2006. [PubMed:16964264] [Full Text]
11. Orsted, D. D., Bojesen, S. E., Tybjaerg-Hansen, A., Nordestgaard, B. G. Tumor suppressor p53 Arg72Pro polymorphism and longevity, cancer survival, and risk of cancer in the general population. J. Exp. Med. 204: 1295-1301, 2007. [PubMed: 17535973] [Full Text]
12. Hancox, R. J., Poulton, R., Welch, D., Olova, N., McLachlan, C. R., Greene, J. M., Sears, M. R., Caspi, A., Moffitt, T. E., Robertson, S. P., Braithwaite, A. W. Accelerated decline in lung function in cigarette smokers is associated with TP53/MDM2 polymorphisms. Hum. Genet. 126: 559-565, 2009. [PubMed: 19521721] [Full Text]
13. Kruger, S., Bier, A., Engel, C., Mangold, E., Pagenstecher, C., von Knebel Doeberitz, M., Holinski-Feder, E., Moeslein, G., Schulmann, K., Plaschke, J., Ruschoff, J., Schackert, H. K., German HNPCC Consortium. The p53 codon 72 variation is associated with the age of onset of hereditary non-polyposis colorectal cancer (HNPCC). J. Med. Genet. 42: 769-773, 2005. [PubMed: 16199549] [Full Text]
13. Ara, S., Lee, P. S. Y., Hansen, M. F., Saya, H. Codon 72 polymorphism of the TP53 gene. Nucleic Acids Res. 18: 4961, 1990. [PubMed: 1975675] [Full Text]
14. Dufour, C., Capasso, M., Svahn, J., Marrone, A., Haupt, R., Bacigalupo, A., Giordani, L., Longoni, D., Pillon, M., Pistorio, A., Di Michele, P., Iori, A. P., Pongiglione, C., Lanciotti, M., Iolascon, A. Homozygosis for (12)CA repeats in the first intron of the human IFN-gamma gene is significantly associated with the risk of aplastic anaemia in Caucasian population. Brit. J. Haemat. 126: 682-685, 2004. [PubMed:15327519] [Full Text]

[redacted] Cadaver Exome Sequencing

[redacted]

Exome Sequencing

- Technique for sequencing all the protein-coding genes in a genome (known as the exome)
  - ~50 million bp (1% of genome)
- Clinically useful and cost-efficient because an exome is a much smaller set of DNA to work with
- Limitations:
  - Cannot detect structural or non-coding variants (epigenetics or intron/noncoding regions) associated with disease
  - Statistical analysis of sequencing data is challenging due to the large volume of data
    - False positive and negative findings due to sequencing, or genetic heterogeneity and population ethnicity will make identification of candidate genes more difficult

Exome Sequencing Technique

- DNA Preparation
  - DNA is fragmented
  - Linker is ligated to sequences of the exome
  - Exome sequences are amplified by PCR
  - Hybridized on capture array
  - Sequenced by fluorescence
- DNA Quality
  - Each base pair is automatically checked during sequencing and scored for quality

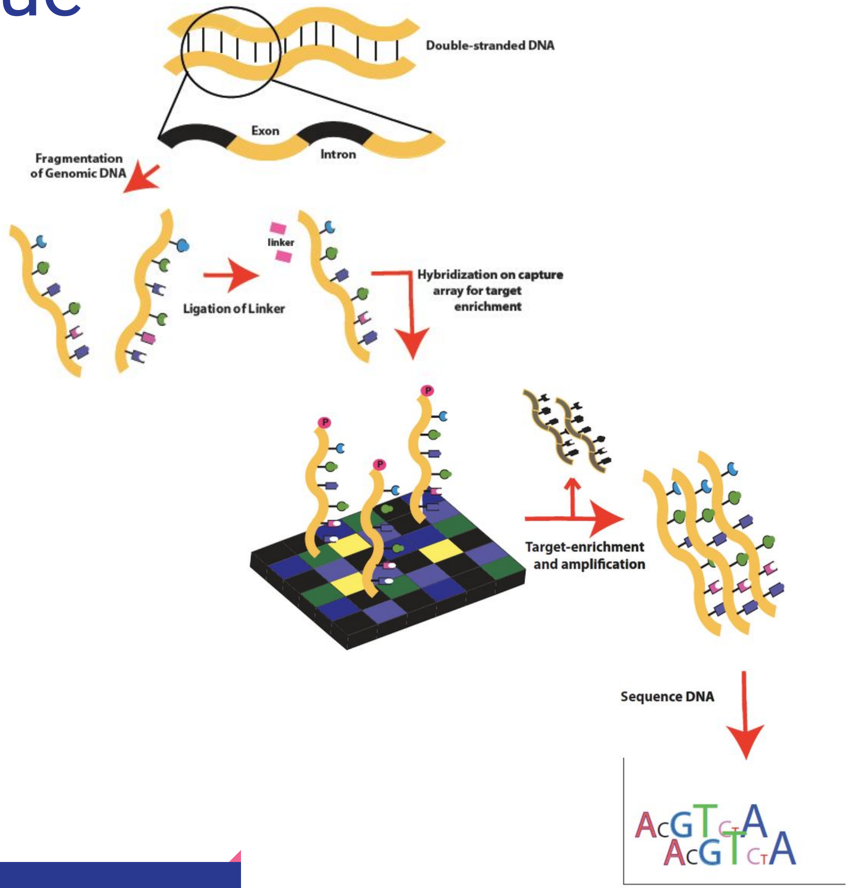

Our Patient

- 89 year-old female
- Ethnicity: Caucasian
- Occupation: homemaker
- Cause of death: Dementia
- Additional observations: pale skin and light gray hair

SNV rs1799990

- Heterozygous for missense A-->G mutation in PRNP gene on chromosome 20
- Met129Val substitution in protein product
- Global Minor Allele Frequency (MAF): 0.2666 (26.66%)
- Affects PRNP protein
- Encodes a membrane glycoprotein that acts as a cell-surface receptor and may transduce a number of neurotoxic signals

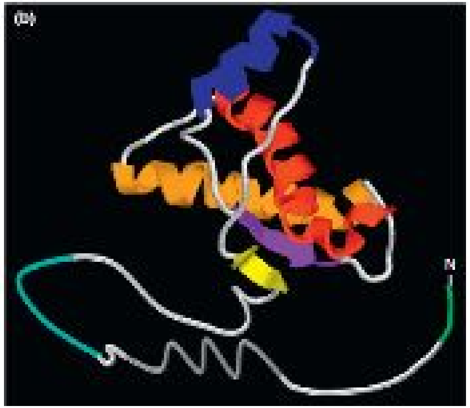

Phenotypes Associated with PRNP Variation

- **Creutzfeldt-Jakob disease**: prion disease involving rapidly progressive dementia and irregular brain waves when measured with electroencephalogram (EEG)
- **Cerebral amyloid angiopathy**: form of angiopathy in which amyloid deposits form in the walls of the blood vessels of the central nervous system; commonly occurs in patients with Alzheimer’s

Cause of death of our cadaver was dementia, so this variation in this gene was of interest; however, it is unknown if this variation had a pathological effect.

SNV rs41292677

- Affects *ABCA4* by causing a GC transversion mutation resulting in Val2050Leu on chromosome 1 (heterozygous)
  - Both of the amino acids possible at this position are hydrophobic branched chain amino acids, but leucine is slightly bulkier than valine residue
- Global MAF: 0.0028/14 (.28%)
- ABCA4 which codes for an ATP-binding cassette (ABC) superfamily transmembrane protein expressed exclusively in retinal cells
  - Genes whose products are transmembrane proteins involved in energy-dependent transport of substrates across membranes
- ABCA4 gene contains at least 50 exons and spans an estimated 150 kB

Phenotypes Associated with ABCA4 Variation

- **Stargardt disease** (fundus flavimaculatus): an inherited form of juvenile macular degeneration that causes progressive vision loss usually to the point of legal blindness
  - Autosomal recessive inheritance

SNV rs16891982 & rs26722

- Affects *SLC45A2*
  - SNV rs16891982: missense mutation (TTC → TTG), resulting in Phe374Leu on Chr. 5 (heterozygous)
  - SNV rs26722: missense mutation (GAG → AAG), resulting in Glu272Lys on Chr. 5 (heterozygous)
- Global MAF
  - SNV rs16891982: .27 (27%)
  - SNV rs26722: .1781 (17.81%)
- SLC45A2 encodes a melanocyte differentiation antigen that is expressed in melanoma cells at a high percentage.
- The biochemical effects of this mutation are unknown.

Phenotypes Associated with *SLC45A2* Variation

- Lys272 (along with leu374) is significantly associated with dark skin, hair, and eye color in Caucasians.
- Consistent with patient’s possible hair color and eye color
- The gene, but not these specific mutations, is associated with Oculocutaneous Albinism, Type IV
    - Hypopigmentation of the skin and hair plus the characteristic ocular changes

SNV rs1126809

- Missense mutation of *TYR* that results in Arg402Gln on chromosome 11 (heterozygous)
- Global MAF: 0.0813
    - The SNP is quite common among Caucasians, with an allele frequency of approximately 0.278 (consistent with our patient's profile).
- *TYR* encodes tyrosinase protein, which catalyzes the first 2 steps, and at least 1 subsequent step, in the conversion of tyrosine to melanin
- Mutation results in tyrosinase peptide that is reactive to temperature and subject to endoplasmic reticulum retention, yielding only 25% of the catalytic activity of the wildtype enzyme

Phenotypes Associated with *TYR* Variation

- Oculocutaneous albinism, Type IB
  - Autosomal recessive and heterozygotes are asymptomatic
  - At birth, individuals with OCA1B have white or very light yellow hair that darkens minimally with age, white skin that over time develops some minimal generalized pigment and may tan slightly with judicious sun exposure, and blue irises that darken to green/hazel or light brown/tan with age

SNV rs35264875

- Chromosome 11q13.3 at position 69078931, heterozygous
- Affects *TCPN2* gene
- Global MAF: 0.0996 (0.02%)
- Causes a missense Met484Leu substitution
  - Both non-polar amino acids
- Encodes a two-pore segment channel involved with intracellular regulation of calcium signals (Calcraft et al. 2009)
- Phenotypically, the missense mutation above is significantly shown to be associated with blond hair (Sulem et al. 2008)

SNV rs1805005 (hom)

- Missense mutation of melanocortin 1 receptor (*MC1R*), resulting in Val60Leu on chromosome 16
- Global MAF: 0.0353 (3.53%)
- MC1R is a G-protein coupled receptor that binds to melanocortin hormones, such as ACTH and MSH.
  - Key protein involved in regulating skin and hair color.
  - When activated by MSH, MC1R initiates a cascade signal, causing the melanocyte to produce eumelanin (dark or black)
  - When deactivated, melanocyte produces phaeomelanin (yellow or red)
- Substitution of valine for leucine, both of which are non-polar amino acids of similar size, would not likely have affected MC1R function

Phenotype Associated with MC1R Variation

- An association between the Val60Leu variant of the MC1R gene and blond/light brown hair and/or fair skin
    - May act as a partially penetrant recessive allele (thus the lighter skin and lighter hair, but not super fair skin and red hair)
- With regards to our patient, due to her somewhat fair skin, she most likely had to wear sunscreen for skin protection.
- Her MC1R variant that was NOT one of the variants found to be associated with melanoma and nonmelanoma skin cancer, but she may have had freckles or sun/age spots

SNV rs1801968

- Missense mutation: C to G at position 132,580,901 (Asp216His) on Chromosome 9 (heterozygous)
- Global MAF: 0.0837/419
- Also known as Torsin 1A, this gene codes an ATPase related to Clp protease and the heat shock family. Expressed mainly in the substantia nigra, cerebellum and dentate gyrus.
- Certain mutations in this gene result in torsion dystonia 1, which is an autosomal dominant disorder.

rs1801968 Phenotype

- Recent evidence points towards this SNP being a risk factor/modifier of torsion dystonia 1. Loss of glutamic acid in Torsin A protein causes mutant protein that disrupts communication between neurons.

- Other variations of the Tor1A gene lead to torsion dystonia, a neurodegenerative disorder. This disorder is characterized by painful muscle contractions leading to uncontrolled twisting movement. Generally begins in one limb around the start of puberty and progresses to the rest of the body within the next decade.

http://www.thelancet.com/journals/lancet/article/PIIS0140-6736(70)91848-9/abstract
http://www.ncbi.nlm.nih.gov/pubmed/18519876,20584926,17503336,11523564,9288096

SNV rs35910969

- Chromosome 17 at position 74749174, heterozygous C-->G mutation
- Affects *SLC9A3R1* gene
- Global MAF: 0.0060 (0.60%)
- Causes a missense Leu110Val substitution
  - Both non-polar amino acids
- Encodes a sodium/hydrogen exchanger regulatory cofactor
  - Interacts with and regulates proteins such as G-protein coupled receptor and protein linkers between integral membrane and cytoskeleton proteins

Phenotypes Associated with *SLC9A3R1* Variant

- Hypophosphatemic nephrolithiasis/osteoporosis
  - Idiopathic renal phosphate loss leading to kidney stone formation
  - Bone demineralization
  - Formation of renal calcium stones
- Loss of heterozygosity of *SLC9A3R1* implicated in development of breast cancer

References

Calcraft, Peter J., Margarida Ruas, Zui Pan, Xiaotong Cheng, Abdelilah Arredouani, Xuemei Hao, Jisen Tang, Katja Rietdorf, Lydia Teboul, Kai-Ting Chuang, Peihui Lin, Rui Xiao, Chunbo Wang, Yingmin Zhu, Yakang Lin, Christopher N. Wyatt, John Parrington, Jianjie Ma, A. Mark Evans, Antony Galione, and Michael X. Zhu. "NAADP Mobilizes Calcium from Acidic Organelles through Two-pore Channels." *Nature* 459.7246 (2009): 596-600. Web. 4 Nov. 2015.

Sulem, Patrick, Daniel F. Gudbjartsson, Simon N. Stacey, Agnar Helgason, Thorunn Rafnar, Margret Jakobsdottir, Stacy Steinberg, Sigurjon A. Gudjonsson, Arnar Palsson, Gudmar Thorleifsson, Snæbjörn Pálsson, Bardur Sigurgeirsson, Kristin Thorisdottir, Rafn Ragnarsson, Kristrun R. Benediktsdottir, Katja K. Aben, Sita H. Vermeulen, Alisa M. Goldstein, Margaret A. Tucker, Lambertus A. Kiemeney, Jon H. Olafsson, Jeffrey Gulcher, Augustine Kong, Unnur Thorsteinsdottir, and Kari Stefansson. "Two Newly Identified Genetic Determinants of Pigmentation in Europeans." *Nature Genetics Nat Genet* 40.7 (2008): 835-37. Web. 4 Nov. 2015.
